# Identifying sustainability priorities among value chain actors in artisanal common octopus fisheries

**DOI:** 10.1007/s11160-023-09768-5

**Published:** 2023-03-04

**Authors:** Gillian B. Ainsworth, Pablo Pita, Cristina Pita, Katina Roumbedakis, Graham J. Pierce, Catherine Longo, Gregory Verutes, Tereza Fonseca, Daniela Castelo, Carlos Montero-Castaño, Julio Valeiras, Francisco Rocha, Laura García-de-la-Fuente, Jose Luis Acuña, M. del Pino Fernández Rueda, Alberto Garazo Fabregat, Alberto Martín-Aristín, Sebastián Villasante

**Affiliations:** 1 Faculty of Business Administration and Management, University of Santiago de Compostela, Santiago de Compostela, Spain; 2 Department of Applied Economics, CRETUS, University of Santiago de Compostela, Santiago de Compostela, Spain; 3 International Institute for Environment and Development (IIED), London, UK; 4 CESAM - Centre for Environmental and Marine Studies, Department of Environment and Planning, University of Aveiro, Aveiro, Portugal; 5 Instituto de Investigaciones Marinas (CSIC), Vigo, Spain; 6 Marine Stewardship Council (MSC), London, UK; 7 Instituto Español de Oceanografía, Madrid, Spain; 8 Departamento de Ecología y Biología Animal, Universidade de Vigo. BA2, Campus de Vigo As Lagoas-Marcosende, 36310 Vigo, Spain; 9 INDUROT, Universidad de Oviedo, Oviedo, Spain; 10 OMA, Universidad de Oviedo, Oviedo, Spain; 11 Centro de Experimentación Pesquera, Consejería de Medio Rural y Cohesión Territorial del Principado de Asturias, Gijón, Spain; 12 Marine Stewardship Council (MSC), Madrid, Spain

**Keywords:** Ecological, Economic, Ethical, Institutional, *Octopus vulgaris*, Social value, Technological

## Abstract

The United Nations (UN) Decade of Ocean Science highlights a need to improve the way in which scientific results effectively inform action and policies regarding the ocean. Our research contributes to achieving this goal by identifying practical actions, barriers, stakeholder contributions and resources required to increase the sustainability of activities carried out in the context of artisanal fisheries to meet UN Sustainable Development Goals (SDG) and International Year of Artisanal Fisheries and Aquaculture (IYAFA) Global Action Plan (GAP) Pillar targets. We conducted a novel ‘social value chain analysis’ via a participatory workshop to elicit perspectives of value chain actors and fisheries stakeholders associated with two Spanish artisanal common octopus (*Octopus vulgaris*) fisheries (western Asturias—Marine Stewardship Council [MSC] certified, and Galicia—non-MSC certified) about their priorities regarding sustainable octopus production and commercialization. Our adapted Rapfish sustainability framework emphasised the importance of economic, environmental, ethical, institutional, social, and technological indicators to different actors across the value chain. We mapped participants’ shared sustainability priorities (e.g. integrated fisheries management, knowledge-based management, product traceability) to six Rapfish indicators, seven IYAFA Pillars and twelve SDGs to reveal how our results can inform ocean policy and actions. This identified how certification incentives and other cooperative approaches can facilitate environmental, economic and social sustainability (e.g. value-added products, price premiums for producers, gender inclusive organisations); support IYAFA priority outcomes (raised awareness, strengthened science-policy interface, empowered stakeholders, partnerships); and help to achieve UN SDG targets (e.g. SDG 14.b, SDG 17.17). The results can inform actors, stakeholders and policymakers about how different actors contribute to efforts to achieve the SDGs and how to manage priorities for sustainable actions within artisanal fisheries and their value chains. We recommend inclusive and equitable participatory knowledge transfer and governance platforms as part of the UN Decade of Ocean Science and beyond where participants can create theories of change towards sustainability involving the development of multi-sectoral ocean policies framed at the level of the value chain and supported by appropriate governance structures.

**Graphical Abstract:**

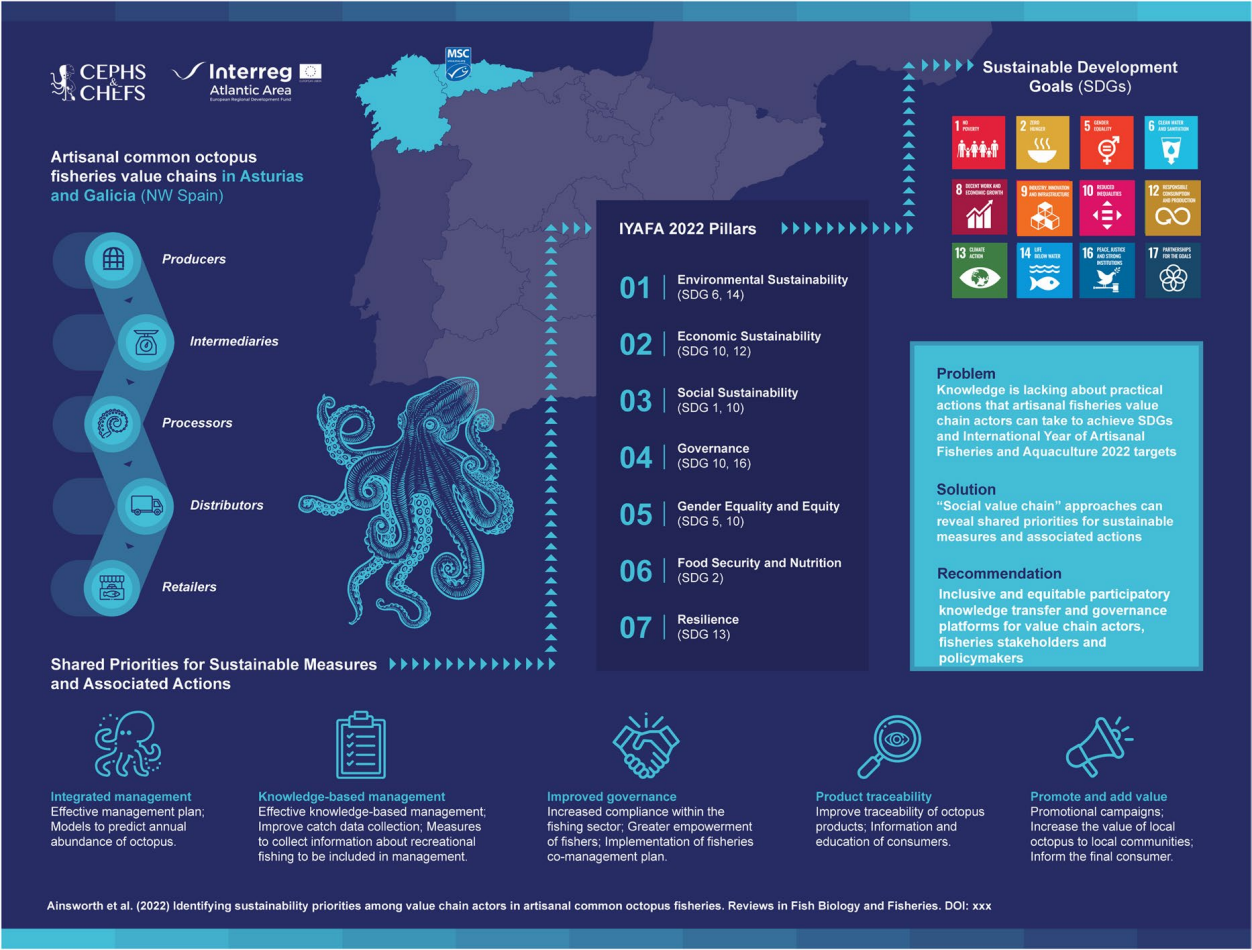

**Supplementary Information:**

The online version contains supplementary material available at 10.1007/s11160-023-09768-5.

## Introduction

The United Nations (UN) Ocean Decade (2021–2030) (United Nations 2021a) highlights a need to improve the way science can quickly inform actions and policy impacts regarding the ocean (Claudet et al. 2020). Relatedly, the ‘International Year of Artisanal Fisheries and Aquaculture’ (IYAFA) aims to recognise and support the contribution of artisanal fisheries and aquaculture to sustainable development and promote dialogue and collaboration between and among artisanal fishery value chain actors (FAO 2022). Actions related to the Ocean Decade and IYAFA aim to recognise some of the major challenges facing artisanal fisheries and draw attention to the sector’s contribution to achieving the UN Sustainable Development Goals (SDGs) (FAO 2022).

A recognised challenge regarding the policies that may promote sustainability of the artisanal fisheries sector and contribute to achieving SDG 14 (Life Below Water) is the translation of policies into practical actions. For example, despite significant reliance by the business sector on maritime resources for inputs and transportation, SDG 14 is rarely identified as a priority for business action (UN Global Compact 2017; Song et al. 2022). Other challenges relate to the inclusion of fishers’ ecological knowledge (FEK) about the local marine environment which can support scientific knowledge and include fishers’ visions of sustainability in management decision-making processes (Freire & García-Allut 1999; Pita et al. 2018, 2020).

Specific trade-related targets for achieving SDG 14 include promoting sustainable fishing, combating illegal, unreported and unregulated fishing (IUU) (SDG 14.4), and providing artisanal fishers with access to marine resources and markets (SDG 14.b). The latter target highlights the need for policies and strategies to empower artisanal fishers to take a more active role in stewardship and management of fisheries resources (UNCTAD 2019, 2021; FAO 2022).

One strategy for identifying and developing practical actions is through stakeholder learning exchanges where ideas can be shared about value addition, product diversification and market development (FAO 2022). A value chain approach within the oceans economy frame can explore stakeholder perspectives about such ideas and provide beneficial environments for all artisanal fisheries stakeholders through value creation that differentiates artisanal fisheries products within lucrative markets (UNCTAD 2019).

Value chains are enhanced supply chains where the aim is to maximise net revenue by adding or creating value to products incrementally along the chain (Bjorndal et al. 2014). Value chain analysis focuses on actors working within the value chain (e.g. producers, processors, retailers) and typically examines economic criteria (e.g. volume, value, price transmission of fisheries products traded). However, only focusing on trade dimensions can mask other key factors such as ecosystem constraints or the sustainability of activities conducted across the value chain (Ospina-Alvarez et al. 2022). To gain a more holistic understanding of how social-ecological factors influence ecological, economic and social sustainability outcomes in the value chains of fisheries, it is essential to also study the social (e.g. non-monetary) values that influence decision-making. We suggest it is critical to understand the values of value chain actors as well as those of stakeholders associated with the fisheries (e.g. fisheries scientists, government regulators, experts from NGOs) who hold different kinds of important knowledge (e.g. local, scientific, FEK) and experience with the system. Therefore, we distinguish between ‘value chain actors’ and ‘fisheries stakeholders’ as sources of different kinds of knowledge and experience.

Artisanal cephalopod (cuttlefish, octopus, squid) fisheries are important sources of seafood (Ospina-Alvarez et al. 2022). For instance, the common octopus (*Octopus vulgaris*) is the most important commercially harvested octopus species in Europe and has considerable social and economic importance especially for southern European countries (Pascual Fernández et al. 2020; Pita et al. 2021). Cephalopod stocks are growing in importance as a global commodity for human consumption, with production having more than tripled over the last 50 years to around 374, 200 tonnes in 2020 (FishStatJ 2020). However, global annual landings are volatile, strongly linked to a few stocks and peaked in 2015 (Moustahfid et al. 2021; Roa-Ureta et al. 2021; Arkhipkin et al 2022). Cephalopods also provide essential ecosystem services and support other fisheries (e.g. as prey), therefore sustainable stock management is becoming increasingly urgent (Hunsicker 2010; Essington & Munch 2014; Arkhipkin et al. 2022).

Sustainability of fisheries has been broadly defined as including the status of the exploited stock, environmental impacts of the fishing activity, efficiency of the management system, and animal welfare as well as consideration of human health, food security, traceability of products, the cultural value of fishing and the social and economic benefits provided by the fishery in terms of income and employment (MSC 2015; Roumbedakis et al. 2021a). Some of these attributes (among others) could also apply to the sustainable management of inter-linked businesses and activities conducted throughout cephalopod fishery value chains, although no studies appear to have examined this yet. Artisanal cephalopod value chains typically differ from those associated with other types of fisheries. For example, they tend to include a horizontal chain with large numbers of actors (as opposed to a vertical chain where a single company owns suppliers, distributors, retail locations etc.), income inequalities are found across the chain, and fishers tend to fare worse in terms of decision-making and economic power than other types of actors (Ainsworth et al. 2023).

Spain is a leader in the global cephalopod trade network and an essential, central player in octopus trade (Ospina-Alvarez et al. 2022), being the main importer of octopus in the European Union (EU) (mostly frozen products originating in Morocco, Mauritania, Portugal and Senegal), and the main exporter of extra-EU frozen products (mostly to the United States) (European Commission 2020). Spain is also one of four major EU producers of octopus, with common octopus comprising almost half of the catch (European Commission 2020).

Two artisanal common octopus fisheries operating in the neighbouring regions of Asturias and Galicia (northwest Spain) are significant. The Asturian fishing sector is highly dependent on artisanal fisheries, whereas Galician fisheries operate on a much larger scale, Galicia being one of the most important fishing regions in the Atlantic area in terms of income and employment (e.g. fishing, fish processing) (Salz & Macfadyen 2007; Villasante et al. 2021a). The common octopus is one of the most important marine invertebrates in these two regions’ fishing traditions and cultures (Fernández-Rueda & García-Flórez 2007; García-de-la-Fuente et al. 2013; 2020; Bañón 2014; González-Álvarez et al. 2016 Pascual Fernández et al. 2020). Since 2000, the western Asturias traditional octopus trap fishery has been managed under a regional Octopus Management Plan characterised by several management measures for sustainability and scientific monitoring (among others) which formed the basis for part of that fleet obtaining MSC certification in 2016 (García-de-la-Fuente et al. 2020).

Important socio-economic aspects (e.g. volume, value) of the value chains associated with Asturian and Galician common octopus fisheries have been described as part of a broader analysis of market opportunities for cephalopods in the Atlantic Area (Roumbedakis et al. 2021b). However, social values underlying value chain actors’ and fisheries stakeholders’ perspectives and decision-making regarding sustainability of activities conducted within these value chains have not been studied previously.

We therefore gathered social values held by value chain actors and fisheries stakeholders from the common octopus fishery value chains in western Asturias (Marine Stewardship Council [MSC] certified) and Galicia (non-MSC certified). We aimed to explore priorities for actions that could increase the sustainability of activities carried out by different value chain actors, and perceptions about impacts of octopus imports and product eco-labelling on the common octopus value chain. Since this was the first time such an investigation had taken place with these two fisheries and their value chains, our overall aim was to learn about important issues rather than compare perspectives about them. Therefore a single workshop was deemed appropriate.

Our objectives were to (a) identify participants’ priorities for sustainable actions, (b) better understand value creation for octopus products and interactions between different activities within the value chain, (c) provide a means for representatives of value chain actors to network and promote collaboration across the value chains, and d) link participants’ shared priorities to relevant Rapfish sustainability evaluation criteria, and to SDGs through the IYAFA GAP Pillars. Participants’ opinions are contextualised with findings from a comprehensive literature review about the two fishery value chains. The outcomes can inform value chain actors, fisheries stakeholders, scientists and policymakers about sustainability priorities for artisanal fishery value chains, as well as actions and policy impacts to help achieve ocean-related UN SDGs.

## Methods

### Common octopus case study areas

**Fig. 1:**
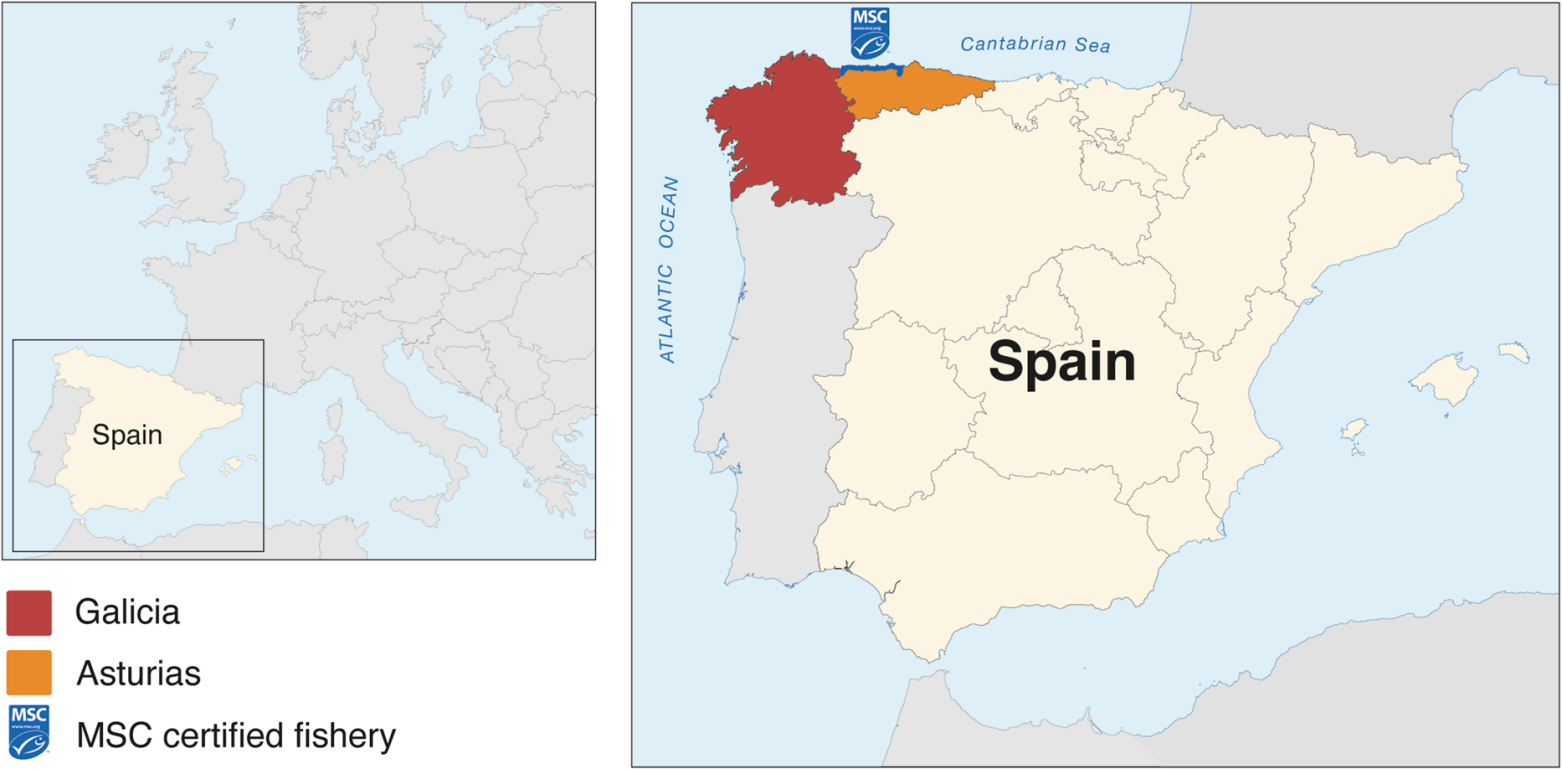
Map of the Asturian and Galician case study areas. The MSC certified artisanal common octopus fishery operates in inner waters along the western coast of Asturias as indicated by the blue shading. The Galician artisanal common octopus fishery operates in inner waters around the entire Galician coastline

Both Asturias and Galicia are Spanish coastal regions located in the Cantabrian Sea and the Atlantic Ocean (Region IV Bay of Biscay and Iberian Coast, North East Atlantic Ocean, FAO 27) (UNEP 2022) (Fig. 1). The common octopus is a benthic cephalopod that occurs in these regions on rocky, sandy and muddy bottoms from the coastline to < 200 m from the edge of the continental shelf (Mangold 1983). It is mostly exploited in these regions by artisanal coastal fleets (Otero et al. 2005; Fernández-Rueda & García-Flórez 2007). The Spanish trawling fleet catches common octopus in the Saharan bank (Northwest Africa) (Balguerias et al. 2002) which is landed and sold in Galician ports.

The Spanish National Government regulates territorial waters and natural resources in the exclusive economic zone and the continental shelf (United Nations 2021b). This includes management of an offshore trawling fleet that catches octopus in the mixed coastal fishery although the current legal minimum mesh size and codend configurations for demersal trawling may not be suitable for regulating fishing of octopus species to comply with minimum landing weight rules (Tosunoğlu et al. 2009). Under the European Union Common Fisheries Policy (CFP), management of common octopus is excluded from quota regulations and is delegated to the regional governments of Asturias and Galicia (Bañón et al. 2018; Villasante et al. 2021a). The regions have powers over fisheries in inshore waters and over shellfish fishing gears in all waters, whereby regional management, monitoring and control of octopus fisheries within Asturian and Galician waters is overseen by the Government of the Principality of Asturias and the Galician Autonomous Government (Xunta de Galicia) respectively (Bañón et al. 2018). Fishery managers recognise the need for better coordination and harmonisation of management measures between the Spanish, Asturian and Galician governments involved (Surís-Regueiro & Santiago 2014; Villasante et al. 2015; Bañón et al. 2018).

#### Western Asturias

The MSC certified western Asturias artisanal octopus fishery operates between the Ria del Eo and the Ria de San Esteban de Pravia (http://pulpodeasturias.es/index.html) on the west coast of Asturias. In this fishery, common octopus is caught using traps and is one of seven species that make up 66% of seafood landings by local artisanal fleets (García-de-la-Fuente et al. 2013; 2020; Bureau Veritas 2021). Fleets are characterised by small family-owned and -operated vessels involving few fishers who live in coastal communities and share the net income (García-de-la-Fuente et al. 2013). Octopus fishing in the certified fishery is regulated through a Management Plan. This plan has been gathering landings and biological data for two decades; it dictates seasonal closures (usually between mid-July and mid-December), minimum landing weight (1 kg), and limits trap numbers and maximum quotas per vessel, among other regulations. Since 2016/2017, a Western Asturias Octopus Monitoring Committee has met twice per year, and the fishermen and administration, with the participation of fisheries scientists (including some of this study’s authors) and experts from NGOs, discuss management measures before they are formalised (FernándezSánchez et al. 2020; García-de-la-Fuente et al. 2020).

A stock evaluation for the western Asturian octopus fishery is now available due to the detailed landings data produced by the co-management plan (Roa-Ureta et al. 2021). Thus, Harvest Control Rules are not set by a simple precautionary principle (as previously) but by objective application of a population dynamics model which is updated every year (Principado de Asturias 2021). This achievement has been possible due to close collaboration between the University of Oviedo, the fisheries administration of the Principality of Asturias and an expert in population modelling (Roa-Ureta et al. 2021).

The octopus has always been a catch of medium value for the artisanal fleet of western Asturias compared to other target species and métiers (González-Álvarez et al. 2016). In 2016, the Navia-Porcía fleet became the first octopus fishery in the world to earn MSC certification along with the right to show the MSC eco-label on its products (Rocliffe & Martin 2020). Certification of the fleet was crucially supported by the Rural Development Group (RDG—a fisheries local action group [FLAG]) Ceder Navia-Porcía which played a fundamental role in organising and engaging the fishers, and facilitating funds awarded for the purpose from Axis 4 of the European Fisheries Fund (FARNET 2017).

Since certification, the fishery has delivered various economic, social and environmental benefits including higher relative prices for fishers (Fernández Sánchez et al. 2020), new markets, better governance (e.g. raising awareness among fishers about the positive role of science in fisheries management) and improved stock health (Rocliffe & Martin 2020). Certified octopus are sold in local auctions, then some are directly exported via traders to markets where the demand for eco-labelled fish products is high (e.g. Northern Europe, USA, Spain and Switzerland) (European Commission 2020; Rocliffe & Martin 2020). Consequently, this fishery commands price premiums up to 25% over uncertified octopus products while publicising the importance of eco-labelling for promoting sustainability and demonstrating that administrative costs relating to certification can be compensated (Fernández Sánchez et al. 2020).

#### Galicia

Fishing for common octopus in Galicia occurs all along the coastline with most landings occurring in the ports of Bueu, Burela, A Coruña, Malpica and Ribeira (Bañón et al. 2018). Fishing is typically conducted by family businesses but includes artisanal fleets using traps in inner maritime (territorial) waters. The artisanal fishery is regulated according to gear type, operating procedures, use of traps, minimum landing weight (1 kg), and maximum daily catches, has an annual closure period usually between May to June and there is a management plan that varies seasonally, along the coast and according to vessel size (Bañón et al. 2018; Pita et al. 2016; Villasante et al. 2016).

A Monitoring Committee of the Annual Octopus Management Plan, consisting of representatives of the fishing sector, scientists, civil society (e.g. WWF) and two administrations (State and Autonomous Community), contributes to annual regulation of this plan (Villasante et al. 2016; Pascual-Fernández et al. 2020). Plans can be modified according to stock status (which is not formally evaluated) in co-management with local fishers due to their knowledge about octopus habitat and biology (Bañón et al. 2018; Pascual-Fernández et al. 2020).

Common octopus populations undergo natural year-to-year fluctuations due to a combination of intrinsic properties of the populations and variation in environmental conditions, e.g. upwelling conditions or changes in salinity linked to rainfall and river discharges (Otero et al. 2008; Roa-Ureta et al. 2021; Robin et al. 2021). Nevertheless, a study of 50 years of data indicates that landings in Galicia have decreased in recent years, which has been attributed to overfishing, habitat loss, lower food availability, ocean warming and pollution (Villasante et al. 2015; Pita et al. 2020). In 2020, an unprecedented reduction of octopus in Galicia significantly affected local prices, income and supply, and was mainly attributed to factors related to climate change (Burgen 2020; Pesca de Galicia 2021; Villasante et al. 2021a). The Galician octopus fishery in Lugo province completed MSC pre-certification assessment processes in 2019 but was considered to have significant weaknesses compared with the level of sustainability required by the MSC Fisheries Standard (Borges & Revenga 2019).

### Understanding sustainability priorities, activities and interactions within the value chain

To fulfil objectives a, b, and c we held a one-day participatory workshop (March 2020, Santiago de Compostela, Galicia)—an appropriate method for reaching people’s underlying values about a topic, facilitating engagement of views among participants, and for informing forward-oriented processes such as organisational change (Burchardt 2013). The workshop was organised by the University of Santiago de Compostela, funded by the Cephs and Chefs project, and co-designed and moderated by a professional facilitator.

We conducted a stakeholder analysis based on the authors’ local knowledge, previous socio-economic value chain analysis conducted with key actors in the region (Roumbedakis et al. 2021b), and descriptions of existing artisanal fishery value chains (e.g. Burch & Maes 2017). This identified that the western Asturian and Galician common octopus fishery value chains included actors from the following sectors: production (e.g. harvesting sector), intermediary (e.g. collection from 1st producer point), processing (e.g. manufacturing, preparation of value-added products), distribution (e.g. distribution/logistics), and retail (e.g. wholesalers, HORECA, fishmongers, supermarkets).

**Fig. 2:**
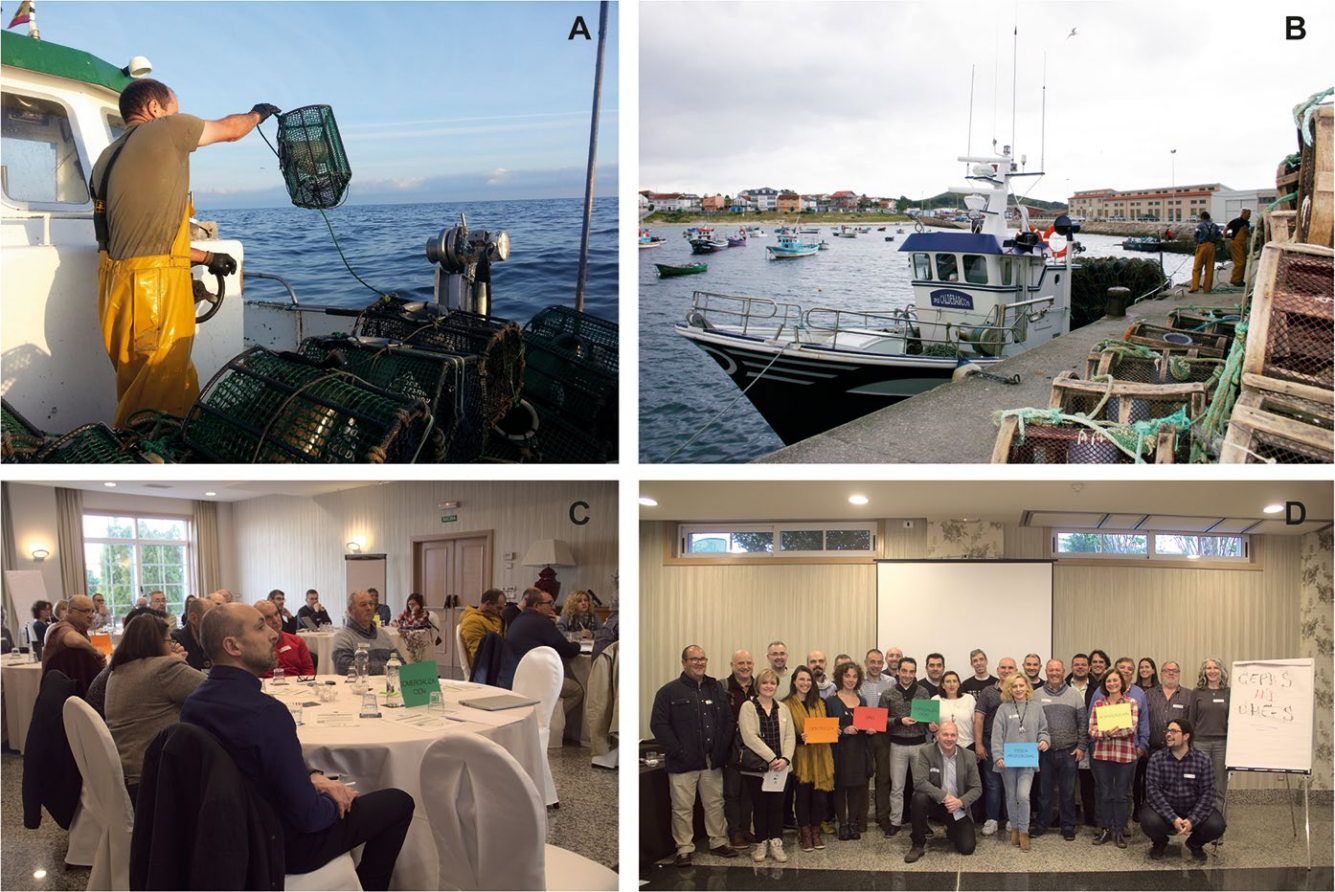
A Fisherman fishing with octopus pots, west coast of Asturias (credit: Centro de Experimentación Pesquera); B: Octopus traps, artisanal vessel and crew, Port of Lira, Os Miñarzos Marine Protected Area, Galicia (credit: Pablo Pita); C: workshop participants working in sectoral groups (credit: Marcela Vazquez Diaz); D: workshop participants and organisers after a day of networking (credit: Marcela Vazquez Diaz)

We invited actors from across the value chain. Representatives from the production, processing and retail sectors agreed to participate, but those from the intermediary, distribution and wholesale sectors did not. Stakeholder representatives from environmental non-government organisations (ENGOs), public administration and scientific communities with expertise on common octopus research and management in the regions also accepted our invitation. In total, 30 participants took part (10 from Asturias): eleven biologists, eight professional fishers, four shipowners, two supermarket owners, two fisheries commercialisation representatives, one economist, one recreational fisher, and one social anthropologist (Table 1; Fig. 2).

**Table 1:** Selected characteristics of the 30 workshop participants (showing number of participants in brackets where more than one)

Sector	Organisation	Region	Role
Business (4)	Association of Shipowners of the Octopus Fishery with Sustainability Certificate (ARPESOS) (2)	Asturias	Commercialisation
	O Sardiñeiro	Galicia	Processor
	Rosa de los Ventos		
ENGO (5)	Marine Stewardship Council	International	Biologist
	Lonxanet Foundation for Sustainable Fisheries	Galicia	Social anthropologist
	WWF	Asturias/Galicia	Biologist
		Madrid	
	Galician Federation of Responsible Maritime Fishing and Nautical Recreation (FEDPEMAR)	Galicia	Recreational fisher
Producer (12)	Secretary General of the Federation of Fishers’ Guilds of Asturias	Asturias	Patron Mayor
	Fishers’ Guild Puerto de la Vega		Professional fisher
	Association of Shipowners of the Octopus Fishery with Sustainability Certificate (ARPESOS) (2)		
	Galician Federation of Fishers’ Guilds	Galicia	Professional fisher
	Fishers’ Guild Bueu		
	Polbo de lLonxa/Pulponor (2)		
	Fishers’ Guild Muros (2)		Shipowner
	Fishers’ Guild Ribeira (2)		
Public administration (5)	Principado de Asturias (Centro de Experimentación Pesquera) (2)	Asturias	Biologist
	Instituto Español de Oceanografía (IEO)	Galicia	Biologist
	Xunta de Galicia (2)		
Scientific (4)	University of Oviedo	Asturias	Biologist
	Universidad Oviedo		Economist
	University of Vigo	Galicia	Biologist
	Spanish National Research Council (CSIC)

Participants included a professional fisher representing one of four member guilds from the MSC certified Navia–Porcia octopus fishery in western Asturias, and a representative from the Association of Shipowners of the Octopus Fishery with Sustainability Certificate (ARPESOS, by its initials in Spanish) which manages the certified fleet belonging to the four guilds and ports included in the certificate (Bureau Veritas 2021). Importantly, Asturian producers’ perspectives represented the MSC certified octopus fishery whereas Galician producer perspectives represented a non-MSC certified octopus fishery.

To begin, we organised participants according to sectoral interests to facilitate constructive dialogue with like-minded people and to encourage knowledge exchange among participants and across regions. Hence, most results represent a consensus between Asturian and Galician participants about both octopus value chains, although we also highlight comments referring specifically to each fishery.

We designed the workshop format based on the authors’ expert knowledge about the octopus fishery value chains and pertinent issues faced by value chain actors (Supplementary file 1: workshop program). First, participants within each sectoral group identified sustainability actions that could be taken within and beyond their own link in the value chain. They wrote individual actions on post-it notes and verbally reported them to everyone. The authors and facilitator collated the post-it notes, sorted the actions by topic and identified several overarching priorities according to these topics which they wrote onto separate post-it notes. They stuck the post-it notes with the priorities on a wall and sorted the associated actions into columns underneath. Participants voted for the three priorities and five actions which were most important to them, and these were ranked in order of votes and sorted according to sectoral groups.

Next, sectoral groups selected one or two priorities each to discuss based on their communal interests, and answered the following questions about them: 1) How could achieving these priorities increase long-term sustainability of the local octopus value chain? 2) What are the main barriers to achieving these priorities? 3) How could different value chain actors contribute to achieving these priorities? 4) What additional resources might be needed? To capture the diversity of views expressed, one person at each table (e.g. the authors) took notes which were agreed by participants before finalising.

Next, we conducted a World Café (The World Café 2015) with participants in mixed groups. Four rounds of collaborative dialogues addressed the following questions relating to the economics and trade of common octopus: 1) How do imported octopus products impact on participants’ businesses? 2) What is the value of sustainable certification or eco-labelling for local octopus products? 3) What potential is there for developing new octopus products or markets for local octopus in Spain and elsewhere? and 4) What are the mechanisms for transmitting the value of local octopus across the value chain? The last question was identified by participants as a sustainability priority in the previous step and due to broad interest in the topic we decided to include it in the World Café discussions. The same note-taking process was implemented as before.

Finally, we asked participants to complete an evaluation survey about the effectiveness of the workshop in achieving certain goals. These included level of satisfaction with the atmosphere of participation and collaboration, and ability to participate and contribute ideas.

### Understanding the Asturian and Galician common octopus fisheries value chains

Rapfish is a multi-disciplinary technique to rapidly evaluate the comparative sustainability of fisheries and is typically conducted by experts assigning quantitative scores to various pre-assigned attributes relating to six indicators for sustainability: ecological, economic, ethical, institutional, social and technological (e.g. Pitcher et al. 2013). We considered this approach to be useful for understanding the current sustainability of the two case study fisheries and providing context for workshop participants’ perspectives. Shortly after our workshop however, Spain entered a state of alarm due to the COVID-19 pandemic and it was unfeasible to conduct an expert opinion evaluation. Instead, we opted to conduct a desktop analysis of relevant scientific documents and value chain company websites which we performed between May 2021 to November 2022. We used an adaptation of the Rapfish framework that we created specifically for this purpose to organise our data, better understand the current sustainability status of the two common octopus fishery value chains and provide context for interpreting the outcomes of our workshop discussions.

We knew from experience that much of the knowledge we sought about the two fisheries was available online in formats other than published and grey literature. Therefore, we sourced articles, reports, theses, websites and statistical databases in English, Galician and Spanish via Google Scholar and internet searches using the terms ‘Asturias’, ‘Galicia’, ‘small-scale’, ‘artisanal’, ‘common octopus’, ‘*Octopus vulgaris*’, ‘fishery’ and ‘certified’ (and their translated equivalents). We also extracted items of interest from the reference lists of scientific publications identified in the search. From the resulting material we rejected irrelevant items that did not provide information relating to one or more of the six Rapfish indicators regarding the two fisheries or other information about their sustainability.

To understand relevant activities of Asturian and Galician common octopus value chain actors, we conducted a concurrent internet search to gather information from company websites in Spanish and Galician using terms and company names based on our knowledge of local value chain enterprises (e.g. ‘Asturias’, ‘Galicia’, ‘common octopus’, ‘*Octopus vulgaris*’, distribut*, process*, retail*, supermarket, Gadis).

### Data analysis

#### Sustainability priorities and actions

First, we counted votes allocated to each sustainability priority and action by different sectoral groups to summarise participants’ preferences. Next, to understand participants’ perspectives, we compiled qualitative data from discussions about each priority according to the sectoral groups voting for each priority. We sorted statements according to topic and sectoral group and treated them even-handedly to present all relevant aspects of the discussions (Stake 2010). Each priority was systematically analysed by examining participants’ responses and identifying similar views so the concepts discussed could be summarised and interpreted according to the context.

#### Implementing the Rapfish framework

We adapted the Rapfish framework to fit an artisanal octopus value chain model by reviewing the sustainability indicators and associated attributes for their relevance to artisanal cephalopod fisheries. We enabled information relating to fisheries and post-production actors to be incorporated and assessed by developing a table to cross-reference information gathered from the desktop analysis according to sectoral actor types from across the Asturian and Galician value chains (e.g. producers, intermediaries) with the six Rapfish indicators (e.g. Ecological) and relevant attributes (e.g. by-catch).

First, we qualitatively reviewed all data collected from the desktop analysis, coded relevant extracts by topic and summarised current knowledge in the table according to the Rapfish indicators and attributes to understand the two fishery value chains and identify important knowledge gaps relating to their sustainability (Supplementary file 2). Extending the review to include company websites enabled us to analyse data about value chain actors not typically represented in value chain analysis and who declined to participate in our workshop (e.g. intermediaries, distributors, wholesalers).

Second, we qualitatively reviewed all data collected from the workshop participants. We organised the data by sectoral group to identify shared and differing perceptions among sectors about sustainability priorities and World Café discussions. Then we thematically mapped this knowledge to relevant sustainability indicator categories in our adapted Rapfish framework.

#### Synthesising knowledge according to Rapfish indicators, IYAFA GAP Pillars and UN SDGs

The IYAFA GAP is structured around seven interconnected Pillars with related outputs and activities designed to stimulate actions that support achievement of nine associated SDGs. The GAP aims to empower small-scale artisanal fisheries and aquaculture and identifies four expected outcomes: ‘raised awareness about small-scale artisanal fisheries and aquaculture to a broad range of audiences’; ‘strengthened science-policy interface by gathering transdisciplinary evidence to support sectoral decision-making and policy processes’; ‘empowered stakeholders through their equal inclusion in decision-making processes’; and ‘development of new and existing partnerships among stakeholders at all levels’.

**Fig. 3:**
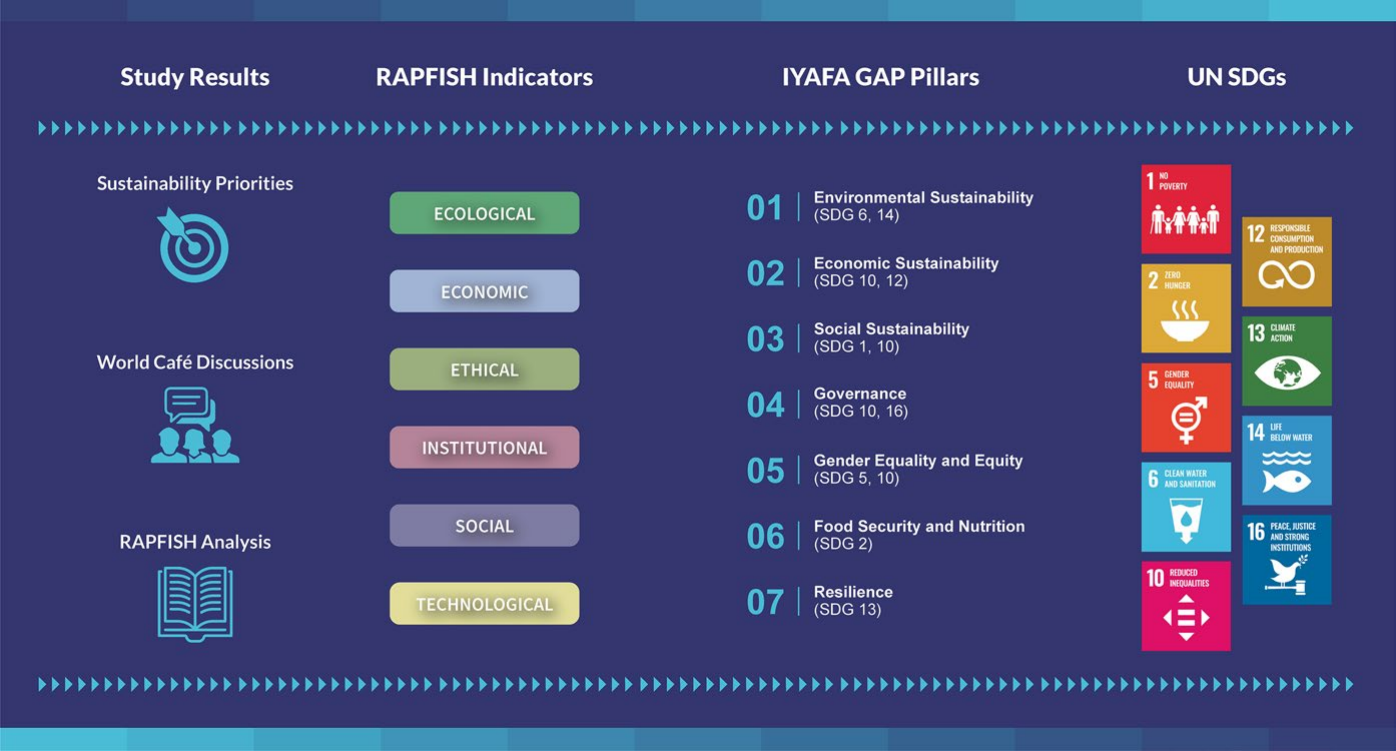
Overview of the process used to develop our key findings. We mapped study results relating to sustainability priorities, World Café discussions and the Rapfish analysis onto relevant Rapfish indicators, IYAFA GAP Pillars and related SDGs. Arrows indicate the direction in which data was mapped (left to right). The SDGs shown are those associated with Pillars in the IYAFA GAP

We recognised that the IYAFA Pillars and associated SDGs provide a valuable framework for organising our findings and discussing how they may contribute to achieving greater sustainability within artisanal fisheries value chains. Therefore, our final analytical step involved drawing upon common themes to synthesise findings regarding participants’ sustainability priorities, World Café discussions and our Rapfish analysis, and mapping these findings by topic to Rapfish indicators, IYAFA GAP Pillars and associated SDGs (Fig. 3).

## Results

### Sustainability priorities and actions

**Fig. 4:**
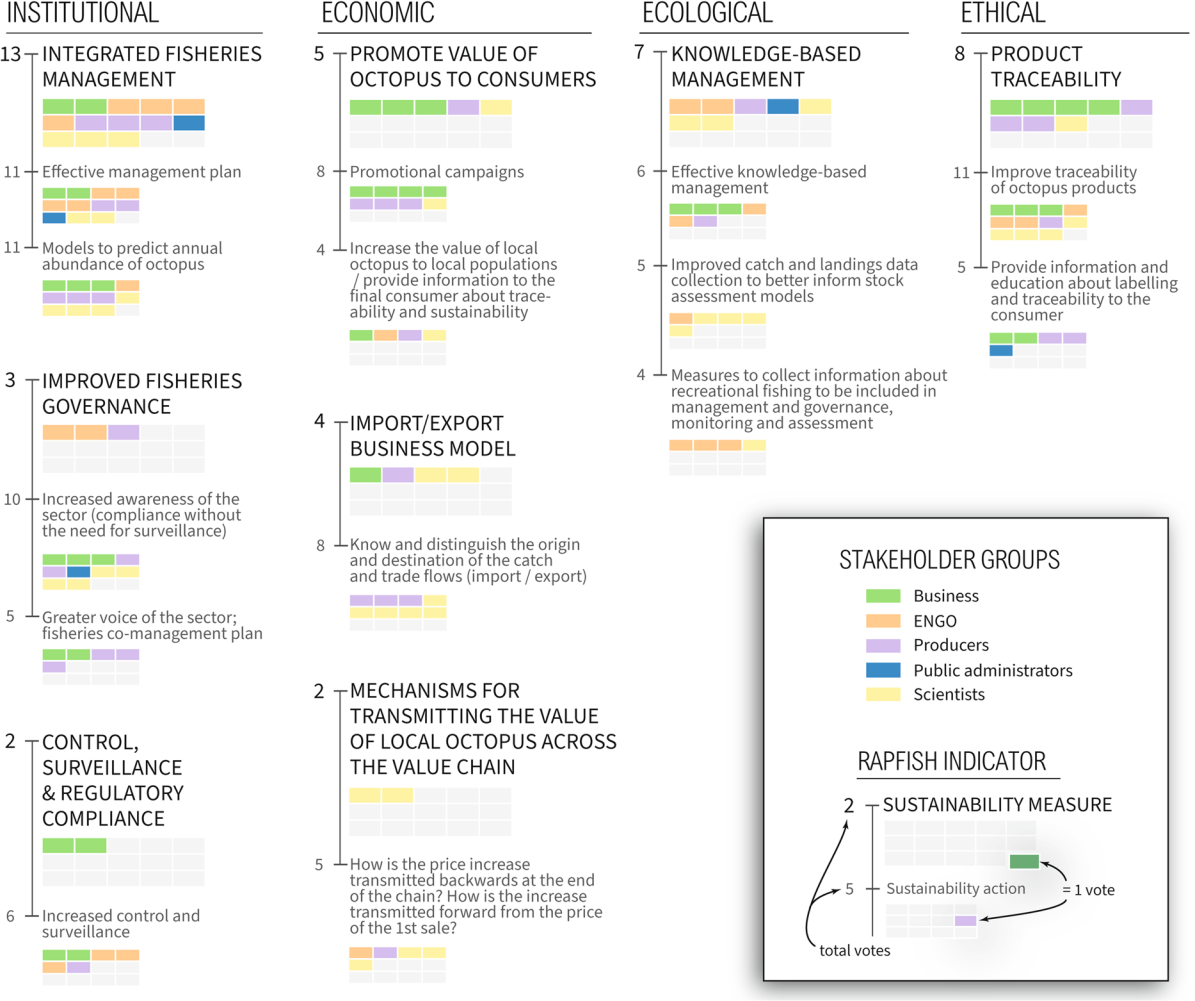
Participants’ shared sustainability priorities and associated actions organised according to four relevant Rapfish indicators for sustainability: institutional, economic, ecological, ethical (Pitcher et al. 2013). Numbers indicate the number of votes given for individual priorities and actions by different sectoral groups, as represented by different coloured boxes

Overall, participants agreed there was an atmosphere of participation and collaboration in the workshop (average score 9.5/10, where a score of 0 = completely disagree and 10 = completely agree) and agreed they could participate and contribute their ideas (9/10). According to participants’ votes, preferences for sustainability priorities and actions differed between sectoral groups (Fig. 4). Participants elected to discuss the following sustainability priorities in depth: integrated fisheries management, improved fisheries governance, knowledge-based management, import–export business model, and product traceability.

### Rapfish analysis

The desktop analysis identified 80 items dated from 2005 to 2022. Of these, we analysed 47 items (17 scientific articles, 15 websites, eight reports, two books, and one: blog, book chapter, government bulletin, legal ruling and thesis).

**Fig. 5:**
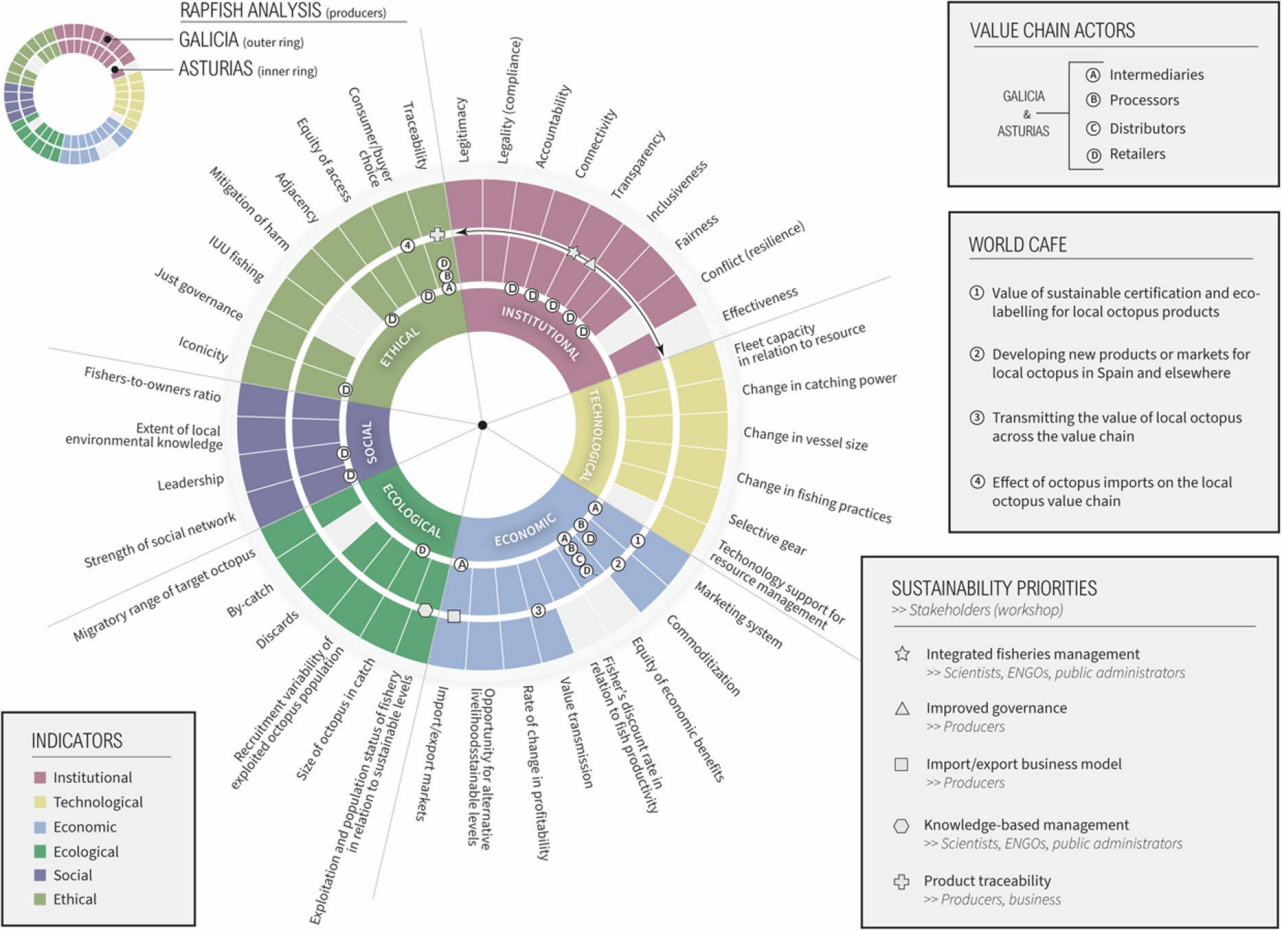
Wheel diagram showing content emerging from the Rapfish analysis and workshop discussions. Coloured cells indicate where data was found relating to Asturian (inner ring) and Galician (outer ring) producers emerging from the Rapfish desktop analysis (grey cells = no data). Letters indicate where data was found relating to other actors in the value chain. Numbers and symbols represent data emerging from the workshop showing how the sustainability priorities and World Café discussions link to individual Rapfish attributes. The arrow in the institutional indicator demonstrates that two sustainability priorities ‘integrated fisheries management’ and ‘improved governance’ corresponded with all institutional attributes

Our adapted ‘artisanal octopus value chain Rapfish framework’ (Fig. 5) differed from the original in that we omitted eight sustainability attributes from the Ecological, Economic, Social and Ethical indicator categories for which we did not find corresponding data. For example, we removed the attribute ‘Intrinsic Vulnerability Index of fish species in the fishery’ from the ‘Ecological’ indicator, because an equivalent index does not exist for cephalopods. We also replaced the word ‘fish’ with ‘octopus’ throughout the attributes as appropriate (e.g. ‘size of octopus in catch’). Additional amendments included adding two new attributes. To the Technological indicator, we added ‘Technology support for resource management’, defined as: availability of technology such as information systems and models, remote sensing, onboard cameras. In the Economic indicator we added ‘Value transmission’, defined as: the distribution of benefits among actors, and amended ‘Export markets’ to ‘Import/export markets’, defined as distinguishing the origin and destination of the catch and trade flows. Finally, we omitted the scoring guidelines which were redundant in our approach and included columns in our table for the following value chain actors: producers (separate columns for Asturias and Galicia), intermediaries, processors, distributors, wholesalers, retailers. Most of the data gathered related to producers (see full results in Supplementary file 2).

Below, we summarise opinions about the five sustainability priorities (SP) participants elected to discuss and perspectives emerging from the World Café discussions (WCD). Each summary is introduced with relevant findings from the Rapfish analysis. In Fig. 5 we show a more complete summary of our data, highlighting knowledge gaps and participant perceptions according to our adapted Rapfish framework.

**Fig. 6:**
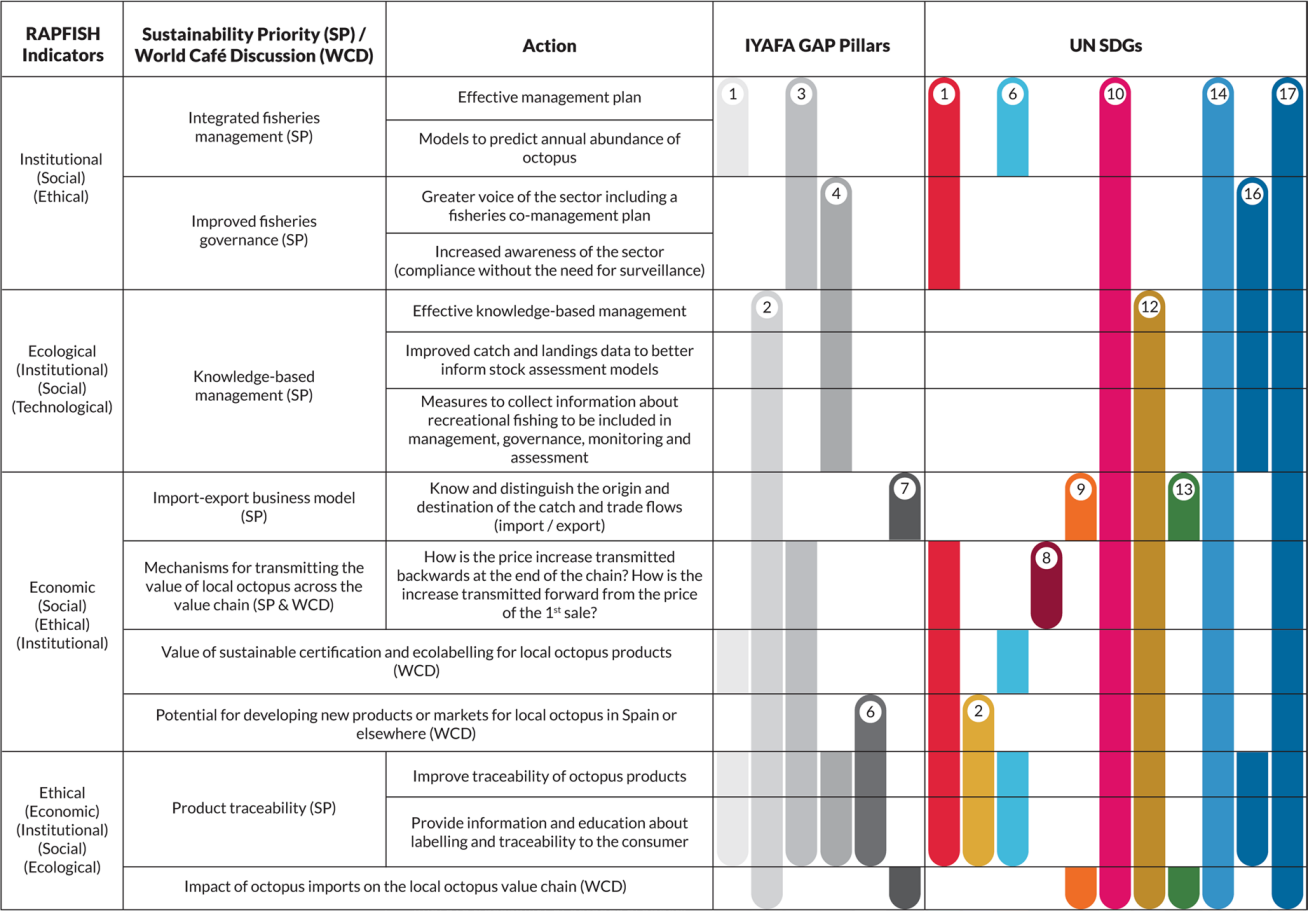
Diagram representing how the results from this study (sustainability priorities [SP] and associated actions, and World Café discussions [WCD]) link conceptually to Rapfish indicators, IYAFA GAP Pillars and SDGs. Data is organised according to four primary Rapfish indicators for sustainability: institutional, ecological, economic and ethical. Numbers in circles indicate which specific IYAFA Gap Pillars and UN SDGs are associated with our data

Mapping our results to the Rapfish indicators revealed that each sustainability priority or World Café discussion topic correlated with one primary indicator, as well as one or more subsidiary indicators, due to the tightly linked and overlapping nature of the themes relating to each indicator and Pillar. Our results are organised below according to the primary Rapfish indicator with which each priority/discussion topic associates most strongly. In Fig. 6 we depict the complex relationships between our results, Rapfish indicators (primary and subsidiary), IYAFA Pillars and SDGs; this includes some connections between Pillars and SDGs not explicitly made in the GAP, but where our results offer useful insights into achieving goal-related targets. For example, we propose that all sustainability priorities contribute to achieving SDG 14.

## Institutional

### Integrated fisheries management (SP)

#### Rapfish analysis

Available data suggest high inter-annual variability of octopus abundance and landings in Galicia likely related to upwelling conditions or other environmental conditions (Otero et al. 2008; Robin et al. 2021). Importantly, the western Asturias and Galician fishing grounds are thought to share a common stock, although this is not reflected currently in the respective regional administrations and offshore national jurisdiction, which currently manage the resource separately (Cabranes et al. 2007; Bureau Veritas 2021).

Fundamental to sustainable fishing for cephalopods and predicting annual abundance is the monitoring–assessment–management cycle. This cycle is dependent upon definition of the stock, full life-cycle monitoring and preferably including the application of ‘depletion methods’ for assessment may be appropriate for some fisheries (Roumbedakis et al. 2021a). Such monitoring and assessment methods have been implemented in Asturias but are not currently in place in Galicia and are urgently needed although they have resource implications. A major obstacle is the definition of octopus stock units to assess conservation and management measures leading to uncertainty regarding stock status and effectiveness of management actions (Rodhouse et al. 2014; Robin et al. 2021).

##### Participants’ perspectives

Scientists, ENGOs and public administrators prioritised integrated fisheries management as a priority sustainable measure. Two associated actions were identified: effective management plan, and models to predict annual abundance of octopus.

Scientists proposed that in this context, integrated management implied similar management rules for all areas that share the octopus resource across western Asturias and Galicia (i.e. there would be no significant difference between Asturias and Galicia regarding regulation of the fishery) which would improve the sustainable management of octopus stocks. They suggested that integrated management could also mean integrating management rules with scientific data, and that management could be adaptive based on other factors as well. Integrated management also meant sharing information across sectors including economists and other fisheries stakeholders.

Public administrators suggested that integrated management would mean fishing effort was matched to abundance based on the results of stock assessments. Integrated management could facilitate dynamic and realistic management. They said that integrated management meant guaranteeing the availability of octopus stock temporally and spatially using real-time assessments and that this might enable the sector to better organise when and how much to fish. They also said integrated management meant access to new or better markets thanks to the improved quality and availability of the product. Finally, integrated management would guarantee the quality of catches by better managing the plans in terms of: implementation of quality controls, establishing certain catch sizes (i.e. size of octopus), and presentation of the product.

Scientists stated that the main impediment to achieving this priority of integrated management was the political frontier between the Asturian and Galician governments. This was described as sometimes interfering with good fisheries management because both jurisdictions have a mandate on their inshore waters, but different management measures can create conflicts among artisanal fishers especially on the border between Asturias and Galicia (e.g. closed seasons are shorter in Galicia than Asturias). Some also explained that offshore and inshore waters fall under different jurisdictions (e.g. the ‘Secretaria General de Pesca’ from the Spanish government controls and regulates offshore trawling while the regional administrations control and regulate artisanal octopus fishing), and that this can be a source of conflict between artisanal and industrial fishers when their fishing grounds overlap. It was said that management of the two fisheries differs since trawling is subject to the CFP due to targeting quota species whereas resource species taken by artisanal fisheries are largely not covered by the CFP which also does not regulate octopus fishing.

Regarding the Galician octopus fishery, ENGOs highlighted that they prioritise implementation of a comprehensive management system that contributes to biological, social and economic sustainability. They identified numerous barriers to achieving these goals, including lack of: discussion and follow-up mechanisms to ensure that management leads to sustainability; transparency in decision making; dialogue mechanisms for all sectors in management; personnel resources or management of existing personnel and financial resources; vision of the sustainable use of resources (e.g. in policy); political will; and culture and tradition of collaboration. These participants proposed that professional and recreational fishermen, scientists, public administrators, first sale buyers and ENGOs could contribute if spaces were created for co-responsibility of all the actors involved in management. However, to be effective this would require the political will to facilitate a paradigm shift to shared resource management as opposed to purely consultation.

Regarding the Galician fishery, public administrators described how integrated management would match fishing effort to abundance based on the results of stock assessments, allow dynamic and realistic management, and guarantee the availability of the octopus product both temporally and spatially. This would give the fishing sector an organisational advantage because they would know when and how much to fish. They suggested that implementing better management plans to guarantee product quality and availability (e.g. establishing quality control, catch sizes and presentation of products) could help with accessing new markets. They identified numerous barriers to achieving this goal, including lack of: sufficient human resources for monitoring and assessment of the fishery (should be increased); collaboration from the Galician fishing sector (should be improved); awareness in the Galician fishing sector, linked to training on sustainability and on the exploitation of natural resources; and citizen training in resource management and marketing. Furthermore, this group identified political and socio-economic interests, IUU fishing in Galicia, and lack of knowledge of the market and the value chain as additional barriers which prevented the octopus products and resources from being more highly valued.

Participants mentioned several important fisheries stakeholders who could contribute to integrated management of the octopus resource: producers who capture and sell octopus (including artisanal, industrial and recreational fishers); scientists who gather and analyse biological, social or economic data relating to octopus management; public administrators and the Member State who oversee regulation of the octopus fisheries; and ENGOs (including those implementing certification programs such as MSC). Participants identified producers, first sale marketers, intermediaries, final distributors, and retailers (including supermarkets and restaurants) as important parts of the octopus value chain.

Public administrators suggested different ways that fisheries stakeholders could contribute: the fishing sector could provide more reliable information and have greater involvement in management (participation and collaboration); public administrators could improve the management and analysis of available data, improve the training of their technicians and collaborate with scientific institutions for data collection and analysis; ENGOs and certification programs or standard owners could raise awareness of good practices, responsible fishing and collaboration; and the business sector could focus more on traceability and guaranteeing the origin of octopus products. Additional resources required would involve improving the personal and material means for monitoring and control through public administration and general education of society in terms of resource management and marketing.

#### Improved fisheries governance (SP)

This sustainability priority was discussed specifically about the Galician octopus fishery.

##### Rapfish analysis

Galician common octopus fishery stakeholders have previously identified several objectives to support long-term management of their fishery, including: increased and ongoing involvement and commitment of the fishing sector (and use of fishers’ traditional ecological knowledge [TEK]) to achieve a co-management plan agreed with the administration which has sustainability of the stock and long-term profitability of the artisanal fleet as its main objectives; increased regularity of Monitoring Committee meetings to discuss new proposals and to empower fishermen, democratise their relations with the administration and prevent potential conflicts; fisheries regulations and management should consider the multi-species and multi-gear nature of the fishery and include updated censuses of vessels actively using each gear (Villasante et. 2021a).

##### Participants’ perspectives

Producers prioritised improved fisheries governance of the Galician octopus fishery as a sustainability measure. They identified two sustainable action priorities: increased awareness of the sector (compliance without the need for surveillance); and greater voice of the sector, including a fisheries co-management plan.

Producers also identified the need to conduct a biological and socio-economic study of the fishery; raise societal awareness about how the fishery is managed; make the fisheries management model more widely known; and apply an evaluation model (in real time) including fishers’ TEK of abundance status of the current and future resource (evolution, captures).

Producers identified several barriers to achieving this priority including a lack of: dynamism by the administration; funding for scientific studies; and sharing management with the sector. It was suggested that different actors in the value chain could contribute by making proposals of interest to the administration. Producers recognised that achieving this priority would require significant additional resources such as: greater funding for scientific studies; greater flexibility and agility (including regular meetings) by design and implementation at the artisanal octopus fishery forum during development of exploitation plans, including recreational fishing; sharing scientific data with the sector and administration; and preparing a report on the development of the octopus campaign.

### Ecological

#### Knowledge-based management (SP)

##### Rapfish analysis

Exploitation of the Asturian certified octopus fishery is currently considered biologically sustainable although the economic return is below sustainable productive potential of the fishery (Robin et al. 2021). The Galician fishery presents a different story where stock status is unknown, there are discrepancies between expected catches and official landings (Villasante et al. 2015; Bañón et al. 2018), and some fishers have expressed concerns about overfishing and other human impacts (Pita et al. 2020). This is despite fishers having previously made urgent calls for priority actions including: updated technology support for resource management, such as information systems and models; the involvement of artisanal fishers to support long-term management of the species; alignment of management measures with biological reference points to avoid long-term reductions in stock productivity; implementation of a two-way exchange of technical information between the fishing sector and the administration, including integrating FEK into management decision-making; changing current technical measures to achieve a more realistic, flexible, and effective control of the octopus fleet effort, health of the stock, and marketing of the product; and improved knowledge of biological aspects of the octopus population (Villasante et al. 2015; Pita et al. 2016).

##### Participants’ perspectives

Scientists, ENGOs and public administrators identified knowledge-based management as a sustainability priority, especially for the Galician fishery. Our definition of this priority recognises the importance of incorporating local (e.g. FEK) and scientific knowledge about the biology and ecology of common octopus, as well as the human and technical aspects of the fishing process into management measures and the monitoring and enforcement of such measures. Three actions were associated with this sustainability priority: effective knowledge-based management (same definition of knowledge as above), improved catch and landings data to better inform stock assessment models, and measures to collect information about recreational fishing to be included in management, governance, monitoring and assessment.

Scientists suggested that adaptive knowledge-based management (with specific and agreed objectives), integrated across regions, sectors, and along the value chain would help with sustainably managing the populations of octopus across Galicia and Asturias. They identified a need for: stock identification, biological data (length–weight relationships, maturity ogives, seasonality of reproduction etc., size, maturity) to set a closed season and identify the recruitment period; better landings data including spatial data on captures since they are not necessarily landed next to capture site); and assessments (e.g. depletion models), predictive models and early in season monitoring of octopus. They also suggested additional resources would be needed to support this work and that a system of key indicators from management could guide prioritisation of data collection. Public administrators suggested that knowledge-based management could improve sustainability of the value chain by enabling better integrated management (see their recommendations for integrated management in Section Institutional).

The scientists identified that a particular challenge regarding biological data was technological support for resource management, including a critical need for data about individual octopus caught (e.g. sex, size) which could inform population abundance models (e.g. generalised depletion models [Roa-Ureta et al. 2021]). ENGOs identified a need for: data on reproduction to adapt closures to biological and economic objectives; updated data on the species’ biological role in the ecosystem; and understanding about how climate change and environmental conditions affect the species. Scientists suggested that models could be used to develop population abundance indices, coupled with monitoring of abundance and linked to management. In this way, scientific data could inform implementation of seasonal closure periods, for example.

A further challenge the scientists and ENGOs identified was incomplete octopus catch and landings, and biological data is required (e.g. continuous data about sexes, sizes, maturity). Scientists explained that available data was based on octopus sales data (e.g. the amount sold in fish market auctions) but since a small proportion of Galician landings did not pass through the official fishing channels these were not declared (e.g. some Galician octopus were sold directly to restaurants or caught by recreational fishers). They said it was not known where all the octopus were caught since there was no mandatory GPS system to monitor where landings came from (although fishers from the western Asturias MSC certified fishery were doing this voluntarily). However, they suggested that data about all catches and landings was more important than knowing where the octopus originated, assuming they are coming from the Asturian or Galician coast due to the geographic limit of artisanal fishing operations. It was explained that Spanish legislation allowed fish and shellfish to be sold in any location, but landings must be declared at the port of disembarkation to obtain a weight label which was essential for transporting the catch.

Scientists identified the main barriers to collecting the data needed on octopus stocks and extent of stocks in the northwest region as: lack of funds; breaching of reporting obligations by some fishers (landings outside markets), enabled by insufficient resources to carry out effective surveillance; natural variability of octopus populations; and politics (e.g. it was explained that the EU subsidised data collection, including data on octopus, but decisions about how and where to sample populations were made at a national level, resulting in sampling gaps). Public administration participants saw difficulties around the impacts of climate change on resource monitoring as a barrier. ENGOs pointed to: an absence of teams working on the collection and interpretation of data; lack of collaboration between the scientific administration and fishing communities and the public; a lack of transparency in the transmission of information between the various sectors and a lack of scientific data in the management plan. These issues presumably did not apply in the western Asturias MSC certified fishery.

Participants agreed that closer collaboration between all actors in general could contribute to achieving this priority. More specifically, scientists suggested that fisheries managers and producers could contribute to gathering better catch and landings data since collecting good data depends on the will of fishers. ENGOs suggested that recreational fishers, scientists, administrators, first sale buyers, and ENGOs could also contribute by providing and interpreting information and through processes and tools for sharing information.

Several kinds of additional resources were said to be required to achieve this goal. For the Galician fishery, scientists and public administrators proposed updated technological support for resource management, including better tracking and on-board monitoring using (new) technology primarily to more accurately record catches (e.g. remote sensing, EMS technology, spatial data, on-board cameras); and implementation of models for data poor fisheries (e.g. the Gómez-Muñoz model) (Comesana & Guerra 2019). For both fisheries, scientists also requested additional funds and improved communication between sectors while ENGOs described a need to create willingness and spaces for all parties to work collaboratively on the priority of knowledge-based management building on common objectives which would need to be defined.

### Economic

#### Import/export business model (SP)

##### Rapfish analysis

According to the literature review, within the western Asturias octopus fishery, certification is thought to have facilitated access to new markets leading to an increase in, and diversification of, demand. Since certification in 2016, 90% of the catch has been destined for export and sold to a Spanish firm specialising in the transformation of octopus which sells the processed product with the MSC label abroad (e.g. the USA) (Fernández Sánchez et al. 2020). Small amounts of octopus were directly exported via traders to markets where the demand for eco-labelled fish products was high (Northern Europe, USA, and Switzerland). The price premium for MSC certified Asturian octopus was said to reach + 20–25% (Fernández Sánchez et al. 2020). Meanwhile, Galicia has a centuries long history of exporting octopus products to other Spanish regions and overseas (e.g. Portugal) (Puig 2012). Furthermore, Galician catches contribute significantly to Spain’s dual roles as landing the greatest amount of cephalopods in Europe (Pascual-Fernández et al. 2020) and being a major international trader in cephalopod products (Ospina-Alvarez et al. 2022).

##### Participants’ perspectives

A sustainability priority for producers was improvement of the seafood trade import/export business model and the associated action was the ability to distinguish the origin and destination of the catch and trade flows (import/export). Producers also recommended that identifying existing sustainability strategies within parts of local and foreign octopus value chains could inform sustainability of the whole local octopus value chain. They identified several key barriers to achieving this priority, including lack of or ignorance about the existence of information and scattered data. Producers suggested that different actors in the value chain could contribute by sharing accurate information from each sector and implementing models that integrate all available data. They suggested that achieving this priority would require interest and commitment from all sectors.

#### Transmitting the value of local octopus across the value chain (SP & WCD)

##### Rapfish analysis

A major sustainability challenge within fishery value chains is equitable distribution of benefits among actors. Product value is either added or created by actors along the value chain and realised from higher prices and/or the development of new markets (e.g. a new product has a competitive advantage over generic products) but is subject to consumer demand, which is driven by a wide range of factors including price, consumer demographics and nutritional content of products (Bjorndal et al. 2014; Villasante et al. 2022). Within fishery value chains, processors and retailers typically receive the most economic benefit from value addition or creation processes due to their bargaining power, while artisanal fishers receive the least and this can lead to adoption of unsustainable fishing practices to increase income (Bjorndal et al. 2014). However, there is also evidence within the western Asturias MSC certified octopus fishery that certification can deliver a price premium when seafood products are eco-labelled and that this premium is obtained across the value chain, including at the production stage (Fernández Sánchez et al. 2020).

##### Participants’ perspectives

In the World Café, participants agreed that transmission of value across the chain was a complex issue due to many influential variables (e.g. fluctuations in the market) but suggested there may be models that integrate large volumes of information such as the General Equilibrium Model for Energy-Economy-Environment (GEM-E3) which could assist with this kind of analysis. Seasonality (inherent in the octopus life cycle) and sharp variations in demand were thought to influence price fluctuation.

Some participants suggested that the value of local octopus is transmitted proportionally throughout the entire value chain, but that not all actors in the chain benefit from the same relative profitability. Actors closest to the distribution and final consumption of octopus products were thought to receive the largest profit margin relative to costs borne. Therefore, some participants proposed that supporting a more equitable and collaborative dialogue between actors at different stages of the value chain is important for seeking a common good or ‘win–win’ solution so that no actor is (dis)advantaged more than another. Maintaining a price that is profitable and that avoids over-exploitation or resource depletion was thought to be appropriate.

Some participants also stated that higher prices for local products could be gained through careful product development, developing marketing messages that communicate the local provenance of the product and emphasise the importance of caring for a fleet which is part of a social and cultural system that needs to be maintained, and through direct sales to hotels or tourists with purchasing power. Thus, participants proposed it is the social responsibility of large final distributors who interact directly with consumers to add value to the octopus resource by selling it at a fair price for all actors across the value chain thereby supporting an ecologically, economically and socially sustainable system.

#### Value of sustainable certification and eco-labelling for local octopus products (WCD)

##### Rapfish analysis

Recent changes in the distribution channels for seafood in Spain have resulted in diverse value chains, prices and margins for seafood due to the ability of retailers and other agents to access products directly from producers and due to the rise of independent wholesale platforms (Bjorndal et al. 2014). In Galicia, landings were sold in an open harbour auction system where buyers include large wholesalers and retail chains, as well local retailers and restaurateurs, meaning that prices differed at each market and were subject to fluctuation (Bjorndal et al. 2014).

Within the western Asturian octopus fishery, certification led to price stability due to the establishment of a future auction system whereby prices were fixed and no longer subject to daily supply and demand (FARNET 2018). Eco-labelling and certification also resulted in economic benefits, perhaps due to the limited supply of MSC certified octopus but increasing world demand. Hence, eco-labelling was likely effective in differentiating seafood products in the market, especially for small or artisanal productions (Fernández Sánchez et al. 2020).

##### Participants’ perspectives

In the World Café, producers from the Asturian MSC certified octopus fishery described how gaining the certification had resulted in several advantages. Importantly, they explained that all the MSC certified octopus was sold in a single batch: therefore fishers could negotiate with buyers to collect the product from local ports and distribute it thereafter, avoiding the need for fishers to transport the octopus to other ports to secure a higher price. Another advantage was that MSC certification had allowed them to create ARPESOS and to agree on a series of rules and measures put in place for attaining certification. The producers said that certification had also resulted in a change of mentality within the Asturian octopus fishing sector (e.g. regarding changes to fishing practices such as using only the permitted number of pots) and fishers’ quality of life was said to have improved along with their relationships with the administration and scientists, and their awareness about sustainability issues. Furthermore, they explained that gaining MSC certification had resulted in their octopus products earning a price premium, but they understood this was not always the case in other MSC certified fisheries.

However, they described MSC certification as having resulted in some disadvantages for local businesses who had less access to local octopus than before certification since octopus prices had risen and certification had shifted traditional sales channels, potentially creating difficulties for some local businesses but advantages for others. Some difficulties in relationships between vessel owners operating inside and outside the MSC certification were noted during the workshop with the suggestion that certification could be extended to other fishing guilds and all landing ports as this would be a ‘win–win’ and the certification itself would gain weight. Relationships have since improved, resulting in another port (Luarca) joining the certification in 2021. An Asturian public administration participant added that collaborating with the fishing sector on the certification process had been a very interesting and positive experience.

#### Developing new products or markets for local octopus in Spain and elsewhere (WCD)

##### Rapfish analysis

Consumption of octopus in Spain was mainly via the hospitality industry, specialised retailers/fishmongers and large-scale retailers (e.g. major supermarket chains) (European Commission 2020). Octopus products included fresh whole raw, whole raw frozen, chilled and thawed, cooked, and processed/value added: marinated, in oil, brine, with garlic, canned etc. Typically, wholesale prices were significantly higher for fresh cooked octopus compared with whole, or thawed and chilled products, while retail prices were higher for products that had undergone some preparation (e.g. whole cooked or cut) than for those that had not (e.g. whole frozen or whole raw) (European Commission 2020).

The process of transforming fresh octopus landed in Galicia and sold frozen in a large Spanish retailer involved wholesaling, thawing, gutting and tenderising the product and included several price transmissions steps: first sale price, auction fees, gutting losses, processing costs (freezing, packaging, transport), processor margin, purchase price for retail platform, transport to retailer, retailer costs and margin, VAT and, finally, retail price—which could be double the first sale price. In comparison, a frozen octopus imported into Galicia from Morocco and sold cooked by a Spanish retailer went through slightly different price transmission steps whereby the retail price may have been three times that of the first sale price (European Commission 2020).

The energy cost and carbon emissions related to fishing and transporting octopus imported into Spain represented an emerging challenge for sustainable cephalopod production and trade (Ospina-Alvarez et al. 2022). Some Galician fishers had already expressed the desire to develop marketing around an easy-to-identify product to encourage consumers to choose it as a local resource from an environmentally sustainable artisanal fishery. One octopus processor stated on their website that they had used octopus by-products in the development of new products, to increase value and promotion of local fishing products, and to favour employment, social inclusion and economic growth of the territory (Rosa de los Vientos 2018).

##### Participants’ perspectives

In the World Café, some participants explained that raw octopus had highest sales in Spain compared with other octopus products, but the market tendency was to demand prepared, pre-cooked products (e.g. cooked octopus, croquettes, sausages). They identified new opportunities for sales as octopus products were reaching new overseas markets where there was low consumption of cephalopods but perceived high demand (e.g. USA, Saudi Arabia). It was suggested that local or domestic consumption of local octopus may be more sustainable from a carbon footprint perspective, than consumption of octopus from further afield. Some participants identified a need for assistance to stimulate local commerce, for example to introduce octopus products into school menus. It was also pointed out that octopus was a seasonal resource since the life cycle was seasonal and there were closed periods when octopus were not harvested (five or six months in western Asturias and one to two months in Galicia), therefore fresh local octopus could not be available on the market all year.

### Ethical

#### Product traceability (SP)

##### Rapfish analysis

A core principle of the MSC Chain of Custody (CoC) Standard was to ‘provide credible assurance that products sold with the MSC ecolabel or trademarks originated from a certified fishery and can be traced through the supply chain to a certified source’, and CoC certified organisations were subject to regular audits (MSC 2019). Therefore, as part of the monitoring and trading phase of the certification process for the western Asturian fishery, MSC CoC certified traders were contacted, a commercial plan for the certified octopus was elaborated, and commercialization tests were carried out (FARNET 2018). The Asturian wholesaler, Asturpesca, has been CoC certified since 2016 in favour of sustainable fishing which covers distribution of common octopus caught with traps by boats belonging to the Tapia de Casariego, Viavelez, Puerta de Vega and Ortiguera cofradías (Asturpesca 2022).

At the time of writing, the Galician government had implemented two certification programs providing hallmarks of quality and provenance for the promotion and defence of Galician products (‘*Galicia Calidade*’ and ‘*pescadeRias*’). Some processing companies had received certification for their octopus products in these programs, indicating the raw materials were of Galician origin or provided added value (Xunta de Galicia 2021a,b). Furthermore, the ‘*Polbo das Rias*’ collective of artisanal fishers guaranteed their octopus products originated from the Galician Rías Baixas region (Polbo das Rias 2021). Selected first buyers and processors also made claims on their websites regarding the traceability of their octopus products to Galician sources (Frigorifico Moldes 2021; Gallego Pereiro 2021; Rosa de los Vientos 2021). Another processor selling common octopus from Spain, Portugal, Morocco and Mauritania claimed to have ‘rigorous traceability control and strict compliance with current National and European consumer information regulations’ (Fesba 2021). The Galician wholesaler Pulponor sells octopus from the cofradías of Finisterre, Corcubion, Lira, Murros, Ou Pindo and Porto do Son under the brand name of ‘Polbo de Lonxa’ which guarantees the origin and quality of the octopus to consumers (Pulponor 2022). Similarly, one major retailer provided product characteristics on its online shopping website including scientific species name, method of production, fishing gear used, capture zone and quality, and sold common octopus from Morocco at the time of writing, as well as products deriving from other octopus species (e.g. lesser octopus *Eledone cirrhosa* from the Bay of Biscay) (Froiz 2021). Another major online retailer sold octopus products which were said to originate in Morocco and Galicia but did not provide any label information (e.g. species name or origin) on its website (Gadisline 2021).

##### Participants’ perspectives

Producers and business actors prioritised product traceability as a sustainability measure. They identified two associated sustainable action priorities: improve traceability of octopus products; and provide information and education about labelling and traceability to consumers.

Producers proposed that traceability of octopus products would be improved by providing consumers with label information about whether the product was of local (e.g. Asturian) or overseas origin for example with a traceability certificate of provenance. Some producers suggested that highlighting traceability through appropriate marketing could help with developing a quality local brand, distinguish producers’ fishing models from other fisheries approaches, and could increase the visibility and distribution of local octopus.

The main barriers to achieving improved traceability of products were said to be business interests of intermediaries, and capture seasons. Producers thought that value chain actors could contribute by labelling products with provenance information while the government could modify regulations for greater transparency to include local origin information. This would require additional resources including greater involvement of the Member State and local entities such as fishing guilds, increased financing to help differentiate the product in dissemination campaigns and involve intermediaries, and greater collaboration between producers and the business sector.

Business participants explained that achieving this priority would increase social-ecological sustainability of the common octopus (with special attention to the socio-economic aspect), increase the value of the product and the local return, and reduce purchasing of immature and ovate octopus due to decreased demand. However, these participants identified many barriers to achieving this goal which related to: the traceability system is incorporated into some labelling (e.g. MSC) but this information does not always reach the consumer; consumer preferences (e.g. low price over sustainability) and consumer inexperience interpreting information on labels which can be unclear (e.g. too much technical information); ineffective advertising campaigns; competition from too many brands and large companies producing products at lower prices, sometimes offshore; lack of government or industry incentives to sell local produce (e.g. educating consumers to demand sustainable local products); and poaching of local octopus.

Business participants suggested several ways that value chain actors could contribute to improved traceability. For example, the administration could support the provision of training courses in commercial areas to explain the differences between local and imported products, provide tax incentives for companies that promote local products, control poaching and increase the minimum catch size in times when juveniles dominate; the administration and wholesalers (e.g. in Asturias) should carry out promotional campaigns using clear labelling to improve consumer understanding; and supermarkets and other retailers could promote local octopus products better and incentivise employees to sell them.

These strategies were said to require additional resources to succeed, including: public and industry financing for coordinated and collaborative advertising campaigns in Galicia and beyond, especially in places without a seafaring culture; effective involvement of the fishery sector in working with the business sector to develop more focused advertising campaigns; private support to promote brands (as in the western Asturias common octopus fishery which is developing a campaign to help pay for the annual MSC certification); in addition to technicians, investment is required to support additional biologists in developing adaptive exploitation plans based on real-time biological data on total catches and/or the mortality caused; closures based on scientific data (e.g. species reproduction) and financial compensation for fishers when biological stoppages are required in the event that fishermen do not have fisheries alternatives; and additional staff in Galicia to control poaching.

#### Impact of octopus imports on the local octopus value chain (WCD)

##### Rapfish analysis

The volume and value of imported octopus products into Spain (especially frozen products) had increased in recent years, with the majority coming from Morocco and Mauritania, but also Portugal and Senegal (Ospina-Alvaerez et al. 2022). Seafood Watch, a major sustainable seafood rating scheme, recommended avoiding the Mauritanian and Moroccan trawl fisheries due to concerns about by-catch of at-risk species and impacts of fishing gear on the marine habitat/substrate (Seafood Watch 2021). Nevertheless, growing external competition from lower-priced imported octopus products from Mauritania and Morocco was considered a threat to Galician artisanal octopus fisheries (European Commission 2020). Although Galician octopus commanded a relatively high price within Spain due to its quality, Moroccan octopus directly competed as it was thought to deliver a better meat yield and ease of cooking and was therefore favoured by restaurants (European Commission 2020). Furthermore, first sale prices of octopus in Spain may have been influenced by changes in landings in Morocco and Spain, and associated demand, while the price of frozen octopus imported into Galicia from Morocco, thawed, gutted and cooked by a Spanish processor, and sold by a Spanish retailer was potentially one third of the retail price of similar Galician products (European Commission 2020).

##### Participants’ perspectives

In the World Café, participants explained there was insufficient local octopus to satisfy existing demand in the region, therefore octopus products were imported at a lower price which had the effect of suppressing local prices. Although some sectors (e.g. retailers, consumers) were thought to want local octopus products and were prepared to pay a price premium for them, most sectors were thought to prefer cheaper options as illustrated by the Asturias certified octopus sales, where the premium was likely also because MSC Chain of Custody certification acts as a guarantee of provenance.

#### Gender inclusive value chains

##### Rapfish analysis

Although not identified specifically as a sustainability priority or World Café discussion, issues of equality, leadership and social responsibility emerged throughout the workshop and were explicitly promoted on the websites of some value chain business. These findings can contribute to achieving IYAFA Pillar 5 Gender Equality and Equity and SDG 5 (Gender Equality).

For example, the Asturian wholesaler Asturpesca claimed to firmly believe in equal opportunities and non-discrimination for reasons of age, gender or country of origin, resulting in a workforce in which diversity was one of its values (Asturpesca 2022). Asturpesca claimed that women accounted for more than 60% of their workforce and led the majority of positions of responsibility in the company while the Management Committee was mostly female (Artupesca 2022).

Similarly, as part of their adaptation strategy after the 1978 Spanish Constitution prohibited gender discrimination, the Galician cofradías claimed to support social welfare, representation and inclusivity with new social values and trends being incorporated into fishing activities and increased professionalism of women in the sector being widely recognised (e.g. as president) (García-Lorenzo et al. 2019; Pascual-Fernández et al. 2020). Within fisheries, the role of women is often as crucial intermediaries and processors, among other things (see e.g. Ainsworth et al. 2023). Furthermore, this essential work is difficult to quantify and still largely goes unnoticed despite changes to the ways such work is recorded in Spain’s official statistics (Herrero-Racionero et al. 2021).

##### Participants’ perspectives

Inclusiveness and equity have been discussed in previous sections, for example equitable distribution of benefits among actors and fairness was mentioned in the section on “Transmitting the value of local octopus across the value chain”. Participants did not identify gender issues as a sustainability priority (or action), and to the best of our knowledge this issue was not discussed in the World Café event.

## Discussion

Our findings provide further evidence that artisanal fisheries face economic power imbalances in value chains, unnecessary barriers to trade, and often lack the appropriate skills and services (e.g. negotiation, marketing) to access markets with healthy products at a fair price (FAO 2022). Furthermore, we demonstrate that artisanal fisheries value chain dynamics can be influenced by broad external forces including international, national and regional fisheries policies and regulations concerning import and export, health and safety and ecolabelling programs for fisheries products. Given the significant global trade in octopus and other cephalopod products, such policies and regulations can affect imports and exports of products sourced from artisanal fisheries around the world. To illustrate, product labelling in Europe must include information about the commercial and scientific name of the species, production method (e.g. caught at sea, type of fishing gear), and catch area (e.g. FAO fishing area). Additionally, fisheries products imported into Europe must be accompanied by an official health certification. Regulation (EC) No 178/2002 established general provisions for traceability whereby manufacturers are required to fulfil requirements regarding food safety procedures and importers must register from where/whom a product was exported (European Parliament & Council of the European Union 2002). European supermarket chains and retailers importing cephalopod products from outside Europe sometimes require that their suppliers are members of credible certification schemes to confirm product safety and quality (Eurofish 2020a). Nevertheless, given the continued growth in online grocery shopping (Nielsen 2015), it is worth noting that our desktop analysis found that this kind of information was often not available via retailers’ shopping websites making it difficult for online grocery shoppers to make informed purchasing decisions regarding octopus products. To combat IUU fishing, catch certificates are demanded from exporters who supply fisheries products to the EU, which are issued by the exporting government, and which must state that the products do not come from IUU fisheries (Eurofish 2020a). In the western Asturias fishery, which exports products to countries around the world (e.g. USA), MSC certification led to improved monitoring, control and surveillance and a review of how fishery certification and CoC certification helped in different ways to improve catch reporting (Longo et al. 2021).

Undergoing the different stages of MSC assessment and certification can improve environmental performance of fisheries including stock status and fishing pressure (e.g. Martin et al. 2012; Gulbrandsen & Hønneland 2014). This can also help address some of the forces described above since certification of the entire supply chain (e.g. through the MSC’s CoC certification) is required to sell products with the MSC label. However, this can be especially challenging when several buyers are involved in a horizontal chain compared with vertically integrated or short supply chains where there is more control over activities and any benefits may be enjoyed by a consolidated company (van Putten et al. 2020). Certification can also lead to greater pricing stability as was found in the Asturian fishery where fishers joined forces in a common organisation, ARPESOS, that allowed them to concentrate, in time and location, the offer from 34 vessels coming from 7 different guilds and thus gained better control of the marketing chain by selling through the future auction system (see also Anderson et al. 2021). From a value chain analysis perspective, this is a major change, considering that fragmentation is a challenge in the artisanal fishing sector globally, and usually has negative impacts in terms of control of the economic variables of first sales, and therefore capitalization of part of the benefits along the value chain. For example, in a Kenyan artisanal octopus fishery, control of the procurement and marketing of octopus by a few actors contributed to high income inequalities between fishers and traders (Wamukota et al. 2014). Similarly, in Fiji and Kiribati, artisanal fishers’ earning varied between 50% and < 10% of the end retail value depending on the species traded, prompting a recommendation for upgrading of value-chain governance through fisher cooperatives or auction systems to improve transparency in pricing and fisher incomes (Purcell et al. 2017).

Consumer interest in rewarding brands that contribute to personal and environmental wellbeing is driving demand for sustainable products and increasing the political and cultural influence of companies that embrace sustainability challenges (Nielsen 2018a). Evidence suggests that MSC certification can generate market differentiation and price premiums for ecolabelled fish and shellfish products which can compensate for the costs of certification and of improvements made by fisheries to enter the program and maintain the certification (e.g. Roheim et al. 2011; Asche & Bronnmann 2017; Fernández Sánchez et al. 2020). Also, despite some evidence that SDG 14 is rarely identified as a priority for business action (UN Global Compact 2017; Song et al. 2022), it is likely that some businesses operating within artisanal fisheries value chains are participating in government programs, fishery initiatives or other value chain actor activities intended to support local livelihoods and promote the provenance of healthy and sustainable resources to consumers, as we found in our study.

Increasing ocean literacy among post-harvest value chain actors and the public could help the business community and consumers to better understand society’s significant reliance on marine resources, and leverage actions to increase sustainability of activities across value chains (Claudet et al. 2020). For example, risks associated with a failure to manage resource depletion (e.g. due to overfishing or environmental variation) can have hard operational consequences on the value chain such as disrupting supply, increasing costs, and diminishing availability of key raw materials (UNEP 2008). Being part of a global value chain means that regional and local fisheries value chains are vulnerable to the effects of global issues, such as the COVID-19 pandemic which created effects including fluctuating supplies, volatile pricing, shifts in product market shares (e.g. from fresh to frozen), breaks in traditional distribution channels and increased uncertainty (Eurofish 2020b; Globefish 2020; Villasante et al. 2021b).

Long-term strategies are required to succeed in the sustainability marketplace. Examples include seeking to build a sustainability strategy to encompass every part of the business and resonating more authentically with consumers by embedding sustainability considerations into each stage of product development, accompanied by finely tuned marketing, distribution and promotions (Nielsen 2018a,b). This approach could equally apply in horizontal fisheries value chains to the development of more cohesive and sustainable business strategies linking producers to consumers. Some of the many advantages of implementing a strategic approach to sustainability include the ability to: develop strong supplier relationships to deliver added-value; ensure reliability; enable innovation and provide sustainable ‘stories’ for communication to consumers to help build brand trust and loyalty; and improve the public reputation and respect for the octopus fisheries and fishers. Investing in sustainability and achieving an ecolabel (e.g. MSC CoC Standard) can lead to increased market value for a company (Luna et al. 2021).

The findings revealed a desire among value chain actors for increased collaboration, communication and traceability across the value chain as well as challenges around achieving strong partnerships (e.g. lack of a multi-sectoral forum to facilitate regular personal engagement and sharing of knowledge between different kinds of sectoral actors). Therefore, in concurrence with the IYAFA aim to empower stakeholders we propose the strategy of implementing regional participatory knowledge transfer and governance platforms where fisheries stakeholders and actors from regional fisheries value chains can share knowledge and information and build interpersonal relationships.

Specific considerations for this kind of stakeholder integration identified in our study which may apply elsewhere include: availability of expertise and funding to carry out required research and capacity building activities; political barriers and frontiers between regions; capacity and willingness to gather, analyse and share data among value chain partners; capacity and resources for improved resource population monitoring and knowledge-based management (e.g. monitoring population fluctuations); IUU; energy cost and carbon footprint associated with the trade of octopus; and climate change. These issues and others could be addressed through the medium of a participatory knowledge transfer and governance platform by supporting the formation of effective partnerships among value chain actors and associated stakeholders and could simultaneously contribute to achieving SDG 17.17 (Encourage and promote effective public, public–private and civil society partnerships, building on the experience and resourcing strategies of partnerships).

We emphasise the importance of including a broad and equitable (e.g. gender, age, cultural background) range of stakeholder representatives in such knowledge transfer and governance platforms. This would help ensure the inclusion and consideration of diverse values and practises related to the value chain (including businesses that rely on fisheries and related maritime resources) and to foster transparent, collaborative solutions identified by participants for achieving mutually agreed sustainability goals (e.g.Zagonari 2008; Artelle et al. 2018). We propose that including the diversity of actors identified in this research (among others) could extend benefits to actors beyond the immediate fisheries context and help with incorporating shared values into policies and processes that apply across the value chain. We also strongly encourage the participation of actors in the platform from the middle of the chain who are typically difficult to engage (e.g. distributors, wholesalers) to ensure their perspectives, activities and sustainability priorities are included in decision-making processes.

Fishing guilds (cofradías) can provide strong leadership within artisanal fisheries since they represent the interests of individual fishing members and communities and are often organised into regional and national federations (Garcia-Lorenzo et al. 2019). Additionally, the support of Rural Development Groups (RDG) can be crucial to artisanal fisheries gaining MSC certification. Hence, there is a clear role for including RDGs, FLAGS and cofradías in knowledge transfer and governance processes. In Galicia, and likely elsewhere, non-fisheries sector professions are not considered a part of artisanal fishing communities (Xunta de Galicia 2014; Garcia-Lorenzo et al. 2019), indicating a need for harmonisation and cohesion of laws and regulations to support sustainability initiatives across different sectors within horizontal fishery value chains.

Finally, given the widespread exclusion of artisanal fisheries organisations from political decision-making processes (e.g. from EU Advisory Councils) (Linke & Jentoft 2016), the inclusion of policymakers in such platforms could elevate their role to that of decentralised science-policy interfaces. Representatives from the scientific, private, public and policy sectors could aim to co-create theories of change towards sustainability involving the development of multi-sectoral policies framed at the level of the value chain and supported by appropriate governance structures. This could potentially result in improved ownership of sustainable practices among value chain actors and the development of science that can more readily support desirable actions (Claudet et al. 2020) as long as such decision-making processes treat all actors in a just and equitable manner, respecting cultural systems, values and knowledge (Arias Schreiber et al. 2022).

## Study limitations

Our research did not aim to compare perceptions about the individual Asturian and Galician fisheries and there were more Galician than Asturian participants (especially among producers). Therefore, although perceptions about priorities for sustainable actions likely differed regarding the two fisheries, and, where possible, we attempted to note these differences when explicitly recorded in the workshop notes (e.g. experiences tied to the MSC certification), discussions represented a consensus among actors and our results are weighted towards Galician issues. Furthermore, a bias towards male over female workshop participants may have precluded discussion about gender issues. Finally, our choice of World Café discussion topics reflects the study authors’ values and priorities based on our understanding of pertinent challenges faced by the fishery value chains at the time, although workshop participants agreed they could participate and share their ideas.

## Conclusions

Our ‘social value chain analysis’ extends the typical value chain analysis concept beyond purely trade concerns to reveal social values underlying actors’ and fisheries stakeholders’ priorities for sustainable actions across the value chain, including practicalities around their implementation. The research exemplifies how the Rapfish sustainability evaluation framework can be adapted to fit artisanal fisheries and their value chains following the modifications we made. We therefore propose that our approach of combining a social value chain analysis with the adapted Rapfish framework may be useful for conducting similar sustainability evaluation processes among artisanal octopus fisheries (or other species groups) and their value chains in other regions and countries. The results can help policymakers to better understand how different actors are currently contributing to efforts to achieve the IYAFA GAP Pillar targets and SDGs (e.g. the business sector) and how to manage priorities for sustainable actions within artisanal fisheries and their value chains.

Identifying shared sustainability priorities of artisanal fishery value chain actors and proposed requirements for achieving those priorities can contribute to a strengthened science-policy interface for example if positive examples of fisheries stakeholders’ sustainable actions were incorporated into responsive legal and policy frameworks relating to governance of fisheries value chains. Highlighting how MSC certification can improve fishers’ quality of life along with their bargaining power within the value chain and the market demonstrates one option of how to empower fisheries stakeholders through co-management practices.

We argue that SDG 17.17 (Encourage and promote effective partnerships) is critical to the successful implementation of all sustainability priorities identified by the participants in our research, and for the successful implementation of sustainability strategies across similar horizontal value chains. We therefore recommend the promotion of and support for participatory knowledge transfer and governance platforms for artisanal fisheries value chain actors and stakeholders as part of the UN Decade of Ocean Science and beyond to improve the way different kinds of knowledge and experience inform action and policies regarding the ocean.

Future research could apply our approach to artisanal fisheries in other parts of the world to examine: gender equality and equity across fishery value chains; the contribution of actors’ activities towards the achievement of SDGs not discussed here (e.g. SDG 3 Good Health and Wellbeing); perspectives of hard to reach value chain actors (e.g. wholesalers, distributors) regarding their sustainability priorities; and gaps in knowledge identified by the Rapfish analysis for some sustainability attributes.

## Supplementary Information

Below is the link to the electronic supplementary material.

Supplementary file1 (PDF 272 KB)

Supplementary file2 (PDF 702 KB)

## Data Availability

All data generated or analysed during this study are included in this published article [and its supplementary information files].
