# Supplementary material for: Identifying sustainability priorities among value chain actors in artisanal common octopus fisheries
Source: Rev Fish Biol Fish. 2023 Mar 4:1–30. Online ahead of print. doi: 10.1007/s11160-023-09768-5 (PMC9985096; doi:10.1007/s11160-023-09768-5)
Supplement: Supplementary file 1 — Supplementary file1 (PDF 272 KB) [file 11160_2023_9768_MOESM1_ESM.pdf]

Identifying sustainability priorities among value chain actors in artisanal common octopus fisheries

Reviews in Fish Biology and Fisheries

Gillian B. Ainsworth[1,2*], Pablo Pita[1,2], Cristina Pita[3,4], Katina Roumbedakis[1,2,4], Graham J Pierce[5], Catherine Longo[6], Gregory Verutes[1,2], Tereza Fonseca[4], Daniela Castelo[4], Carlos Montero-Castaño[6], Julio Valeiras[7], Francisco Rocha[8], Laura García-de-la-Fuente[9], Jose Luis Acuña[10], Mª del Pino Fernández Rueda[11], Alberto Garazo Fabregat[12], Alberto Martín-Aristín[12], Sebastián Villasante[1,2]

[1] Faculty of Business Administration and Management, University of Santiago de Compostela, Santiago de Compostela, Spain

[2] CRETUS, Department of Applied Economics, University of Santiago de Compostela, Santiago de Compostela, Spain

[3] International Institute for Environment and Development (IIED), London, UK

[4] CESAM - Centre for Environmental and Marine Studies, Department of Environment and Planning, University of Aveiro, Aveiro, Portugal

[5] Instituto de Investigaciones Marinas (CSIC), Vigo, Spain

[6] Marine Stewardship Council (MSC), London, UK

[7] Instituto Español de Oceanografía

[8] Universidade de Vigo. BA2, Departamento de Ecología y Biología Animal, Campus de Vigo As Lagoas-Marcosende, 36310 Vigo, Spain

[9] INDUROT, Universidad de Oviedo, Spain

[10] OMA, Universidad de Oviedo, Spain

[11] Centro de Experimentación Pesquera, Consejería de Medio Rural y Cohesión Territorial del Principado de Asturias, Gijón, Spain

[12] Marine Stewardship Council (MSC), Madrid, Spain

*corresponding author: Gillian B. Ainsworth: gill.ainsworth@usc.es;

Adding value to Common octopus across the value chain

Workshop program

Saturday 7th March 2020, Gran Hotel los Abetos, Santiago de Compostela

Times		Details
8:45	9:00	Registration and coffee
9:10	9:30	Organiser welcome, presentation of agenda, presentation of participants
9:30	10:30	Introduction: presentations from the project team (35 min)<br>- Cephs and Chefs project and WP5 overview<br>- Common octopus case study:<br>• Monetary value chain analysis<br>• Non-monetary value chain analysis<br>• Discussion in pairs (10 mins)<br>• Questions and answers (15 mins)
10:30	11:30	• Participant mapping<br>• Identifying sustainability actions<br>• Organisers categorise actions<br>• Feedback on sustainability actions and categories<br>• Voting for preferred actions and categories
11:30	12:00	Break
12:00	13:30	Selection and discussion of top categories in break out groups:<br>• How could achieving this priority increase long term sustainability of the octopus value chain? (20 mins)<br>• What are the main barriers to achieving this priority? (20 mins)<br>• How could different value chain actors contribute? (20 mins)<br>• What additional resources are needed? (20 mins)
13:30	14:15	Break out groups present feedback on their discussion
14:15	15:15	Lunch

Interreg Atlantic Area
European Regional Development Fund
EUROPEAN UNION
CEPHS & CHEFS
