# Supplementary material for: Identifying sustainability priorities among value chain actors in artisanal common octopus fisheries
Source: Rev Fish Biol Fish. 2023 Mar 4:1–30. Online ahead of print. doi: 10.1007/s11160-023-09768-5 (PMC9985096; doi:10.1007/s11160-023-09768-5)
Supplement: Supplementary file 2 — Supplementary file2 (PDF 702 KB) [file 11160_2023_9768_MOESM2_ESM.pdf]

Identifying sustainability priorities among value chain actors in artisanal common octopus fisheries

Reviews in Fish Biology and Fisheries

Gillian B. Ainsworth[1,2*], Pablo Pita[1,2], Cristina Pita[3,4], Katina Roumbedakis[1,2,4], Graham J Pierce[5], Catherine Longo[6], Gregory Verutes[1,2], Tereza Fonseca[4], Daniela Castelo[4], Carlos Montero-Castaño[6], Julio Valeiras[7], Francisco Rocha[8], Laura García-de-la-Fuente[9], Jose Luis Acuña[10], Mª del Pino Fernández Rueda[11], Alberto Garazo Fabregat[12], Alberto Martín-Aristín[12], Sebastián Villasante[1,2]

[1] Faculty of Business Administration and Management, University of Santiago de Compostela, Santiago de Compostela, Spain

[2] CRETUS, Department of Applied Economics, University of Santiago de Compostela, Santiago de Compostela, Spain

[3] International Institute for Environment and Development (IIED), London, UK

[4] CESAM - Centre for Environmental and Marine Studies, Department of Environment and Planning, University of Aveiro, Aveiro, Portugal

[5] Instituto de Investigaciones Marinas (CSIC), Vigo, Spain

[6] Marine Stewardship Council (MSC), London, UK

[7] Instituto Español de Oceanografía

[8] Universidade de Vigo. BA2, Departamento de Ecología y Biología Animal, Campus de Vigo As Lagoas-Marcosende, 36310 Vigo, Spain

[9] INDUROT, Universidad de Oviedo, Spain

[10] OMA, Universidad de Oviedo, Spain

[11] Centro de Experimentación Pesquera, Consejería de Medio Rural y Cohesión Territorial del Principado de Asturias, Gijón, Spain

[12] Marine Stewardship Council (MSC), Madrid, Spain

*corresponding author: Gillian B. Ainsworth: gill.ainsworth@usc.es;

ESM Table 1: Summary of available knowledge about sustainability of the Asturian and Galician common octopus (O. vulgaris) fisheries based on Rapfish sustainability indicators (www.rapfish.org). Value chain columns are based on a diagram produced by Manta Consulting (Burch & Maes 2017). Yellow highlighted text indicates information specifically about the MSC certified Western Asturias common octopus fishery as opposed to the Asturian common octopus fishery in general. The full reference list is included below the table.

**Rapfish evaluation**		**Common octopus value chain (Asturias and Galicia)**					
**Sustainability indicators**	**Attributes**	**Producers**		**Intermediaries (Collection from 1st producer point)**	**Processors (Preparation of value-added products)**	**Distributors (Distribution/logistics)**	**Retailers (Wholesalers, HORECA, fishmongers, supermarkets)**
		**Asturias**	**Galicia**				
(Pitcher et al. 2013)		(Fernández-Rueda et al. 2007; García-de-la-Fuente et al. 2013; González-Álvarez et al. 2016; FARNET. 2018; European Commission 2020; European MSP Platform 2020; Fernández Sánchez et al. 2020; Bureau Veritas 2020; García-de-la-Fuente et al. 2020; Rocliffe & Martin 2020; Principado de Asturias 2021; Roa-Ureta et al. 2021; Robin et al. 2021)	(Otero et al. 2005, 2008; Amigo Dobaño et al. 2009; Puig 2012; European Commission 2013; Bañón 2014; Surís-Regueiro & Santiago 2014; Pita et al. 2016; Villasante et al. 2016; Álvarez Ballesteros 2018; Bañón et al. 2018; Borges & Revenga 2019; García-Lorenzo et al. 2019; European MSP Platform 2020; Pascual-Fernandez et al. 2020; Pesca de Galicia 2021, 2022; Pita et al. 2021; Polbo das Rias 2021; Robin et al. 2021; UNCTAD 2021b; United Nations 2021b; Villasante et al. 2021b; WTO 2021; Xunta de Galicia 2021a,b)	(Bjorndal et al. 2014; Xunta de Galicia 2014; Eumofa 2020; Frigorifico Moldes 2021; Gallego Pereiro 2021; Pesca de Galicia 2021, 2022)	(Xunta de Galicia 2014; European Commission 2020; Fesba 2021; Frigorifico Moldes 2021; Rosa de los Vientos 2018, 2021)	(Xunta de Galicia 2014; European Commission 2020)	(Xunta de Galicia 2014; European Commission 2020; Froiz 2021; Gadisline 2021; Asturpesca 2022; Pulponor 2022)
Ecological evaluation	Exploitation and population status of fishery in relation to sustainable levels	In 2000, the regional fishing authority approved an Octopus Management Plan for this fishery, with the aim to secure environmental (stock) and socioeconomic sustainability (increasing profitability levels and first-sale prices).<br><br>An evaluation conducted by Bureau Veritas prior to MSC certification in 2016 showed that the octopus population of the Asturian west coast was in a healthy situation and that the fishery was well managed, with minimal impact on the marine ecosystem.<br><br>Exploitation is currently considered to be biologically sustainable but economic return is below	Stock status at the Galician level is unknown.<br><br>Overfishing is a key point for the artisanal fisheries segment.<br><br>Need to align management measures with biological reference points to avoid long-term reductions in stock productivity: knowledge of biological aspects of O. vulgaris population is a priority.<br><br>Need to integrate fishers' LEK/TEK into management decision-making: invaluable resource, especially when scientific knowledge is lacking.<br><br>An MSC pre-assessment evaluation of the Lugo province fishery (Arco Ártabro and Arco Cantábrico				

**Rapfish evaluation**		**Common octopus value chain (Asturias and Galicia)**					
**Sustainability indicators**	**Attributes**	**Producers**		**Intermediaries (Collection from 1st producer point)**	**Processors (Preparation of value-added products)**	**Distributors (Distribution/logistics)**	**Retailers (Wholesalers, HORECA, fishmongers, supermarkets)**
		**Asturias**	**Galicia**				
		sustainable productive potential of the fishery.	combined [Galician management zones B&C combined, corresponding to part of ICES division 8.c], northern Galicia) found that the available relative abundance index points to a stable population, however a defined Harvest Control Rule and specific short and long-term management objectives are lacking.				
	Fish life history factors						
	Size of octopus in catch	1kg minimum.	1kg minimum. Some desire to increase legal size to raise market price and income of fishers with fewer impacts on stock.				
	Recruitment variability of exploited octopus population	A stock evaluation for the western Asturian octopus fishery is now available due to the detailed landings data produced by the co-management plan. Thus, Harvest Control Rules are no longer set by a simple precautionary principle (as previously) but by objective application of a population dynamics model which is updated every year. This achievement has been possible due to close collaboration between the University of Oviedo, the fisheries administration of the Principality of Asturias and an expert in population modelling.	Landings and abundance indices show high inter-annual variability which depend on upwelling conditions in Galicia or other environmental parameters like rainfall in the Gulf of Cadiz. Such environmental drivers could be used for short term predictions and in-season management.<br><br>Landings have decreased in recent years likely related to environmental variation in Galician estuaries, pollution, overfishing and ineffective monitoring and control. In the Lugo fishery, monitoring is in place to collect data on catches and biological data, but no independent abundance surveys are carried out.				
	Selective fishing factors						
	Discards	Due to its high value O. vulgaris is rarely discarded and undersized specimens are considered to have a high survival rate when returned to the sea.					
			Approximately 85% of by-catch is discarded and echinoderms account for 98% of discarded species.				

**Rapfish evaluation**		**Common octopus value chain (Asturias and Galicia)**					
**Sustainability indicators**	**Attributes**	**Producers**		**Intermediaries (Collection from 1st producer point)**	**Processors (Preparation of value-added products)**	**Distributors (Distribution/logistics)**	**Retailers (Wholesalers, HORECA, fishmongers, supermarkets)**
		**Asturias**	**Galicia**				
	By-catch		There is a desire among fishers to enforce a limit on the by-catch of octopus by vessels engaged in other coastal fisheries.<br><br>Around 30% of total biomass caught by octopus fishers comprises by-catch including ~180 species and ~40 additional species groups. Approximately 15% of by-catch is commercialised or consumed and the remainder discarded.<br><br>An MSC pre-assessment evaluation of the Lugo province fishery found that the fishery is quite selective with no major by-catch species or impacts in the system. There is also a lack of information regarding bait species and alternative measures to mitigate by-catch mortality.				
	Spatial (geographical) stability factors						
	Migratory range of target octopus	Limited seasonal migrations: it is located in deeper waters during winter and shallower waters during summer					
Technological evaluation	Fleet capacity in relation to resource	Although other vessels can fish in the waters of the certified fishery, only the 27 MSC registered vessels are entitled to use the certification and to enter octopus from the fishery into the four auction ports of Navia-Porcía included in the MSC certificate. There is a limit of 125 pots per fisherman with a maximum of 350 pots per boat.<br><br>Measures to control fishing effort include an annual catch limit of	In 2019, 1217 small-scale vessels were authorised to fish with octopus traps.				

Rapfish evaluation		Common octopus value chain (Asturias and Galicia)					
**Sustainability indicators**	**Attributes**	**Producers**		**Intermediaries (Collection from 1st producer point)**	**Processors (Preparation of value-added products)**	**Distributors (Distribution/logistics)**	**Retailers (Wholesalers, HORECA, fishmongers, supermarkets)**
		**Asturias**	**Galicia**				
		10,000 Kg per vessel and year, a minimum capture weight of 1 kg per specimen caught, and a maximum number of 125 traps per crew member (limit of 350 per vessel). The fishery is operated mainly by small-scale multi-gear boats that catch octopus in shallow waters on fishing trips of a few hours (returning to port on the same day). But during its operating months, this fishery is also able to alternate octopus traps with other fishing métiers.					
	Change in catching power	Both total landings of octopus and the number of boats included in the Octopus Management Plan annually have decreased yearly since 2007 (from 61 vessels in 2007 to 38 vessels in 2014). Some active vessels remain in the fishery each season, while others leave after some years of fishing. Some boats simply fish in certain years (according to their expectations about "how good" the octopus recruitment has been, which is a powerful driver for some vessels to enter the fishery).	Registered vessels decreased progressively at a rate of ~28 vessels per year since 2003. Similarly, the effective license usage in terms of operational licenses and working days diminished at a rate of ~24 vessels and 3726 days per year, respectively, since the maximum reached in 2004. Between 2001 and 2016, the estimated number of traps by year showed decreasing trends concurrent with the reduction in operational fleet metrics.				
	Change in vessel size	Most of the vessels in the Octopus Management Plan are small-scale multi-gear boats (average total length of 9 m in 2007–2014) registered as *multi-gear in the Cantabrian and Northwest fishing ground -CNW6* according to the Spanish Census of Active Fishing Fleet.<br><br>Vessels under the MSC eco-label range are 10-12 meters in length	Between 2001 and 2016, there was a yearly reduction of the lower (type I) and top segments (type V, type VI and VII) in favour of medium segments (types II to IV) especially type III.				

Rapfish evaluation		Common octopus value chain (Asturias and Galicia)					
**Sustainability indicators**	**Attributes**	**Producers**		**Intermediaries (Collection from 1st producer point)**	**Processors (Preparation of value-added products)**	**Distributors (Distribution/logistics)**	**Retailers (Wholesalers, HORECA, fishmongers, supermarkets)**
		**Asturias**	**Galicia**				
	Change in fishing practices	This artisanal fishery based on pots and traps has traditionally relied heavily on workforce and physical strength on-board (the octopus fishery requires enough crew members to cast and haul the traps from the sea and to quickly harvest and re-bait). Traditional ecological knowledge continues to be very important. The only changes occurred in the last years refer to the installation of mechanical haulers on-board of some vessels and the use of artificial bait by part of the fleet in the Octopus Management Plan.	Between 2005 and 2016, fishing effort, in terms of number of vessels that declared fishing activity, showed remarkable seasonal fluctuations with similar patterns in all coastal zones.				
	Selective gear	The artisanal fleet accounts for all O. vulgaris landings, and pots are the only authorised gear.	The artisanal fleet accounts for >90% of O. vulgaris landings in Galicia. Artisanal vessels mostly use octopus traps (80% of catches), as well as crustacean traps, trammel nets, gill nets, and hook and lines. Industrial bottom trawling activities are permitted in areas >100 m depth. The octopus trap is a high selective gear with octopus representing 70.4% of total catch.				
	Technology support for resource management		Gómez-Muñoz model can be used as an independent tool for estimating catch and effort and testing the reliability of landing statistics.				
Economic evaluation	Fishers' discount rate (d) in relation to octopus productivity	The octopus has been a very attractive species for artisanal vessels in the region because it reaches significantly higher first-sale prices than those registered by most of sea products commercialised in the regional fishing guilds. Octopus is a strategic resource for this fleet and for the					

Rapfish evaluation		Common octopus value chain (Asturias and Galicia)					
**Sustainability indicators**	**Attributes**	**Producers**		**Intermediaries (Collection from 1st producer point)**	**Processors (Preparation of value-added products)**	**Distributors (Distribution/logistics)**	**Retailers (Wholesalers, HORECA, fishmongers, supermarkets)**
		**Asturias**	**Galicia**				
		entire region. In terms of landings, octopus catches on the west coast of Asturias accounted for around 77% of total octopus first sales in local fishing guilds of Asturias before the ecolabelling process (between 2007 and 2014).<br><br>Certification has led to stability in prices. The certification has allowed the establishment a "future auction" system. In this procedure, the price is fixed for all the catches made in the following 15 days. The price is no longer subject to the daily supply-demand.					
	Rate of change in profitability	Before MSC certification, the octopus' price in all ports of Asturias was very similar with an average of the annual price around 4.89 €/Kg. After 2015, there was a general increase in market prices of Asturian octopus, although this increase has been more relevant for those ports with the MSC certification. Thus, the average of the annual prices in MSC-certified ports increased up to 8.01 €/Kg versus the average of the rest of the ports in Asturias with an average price of 6.93 €/Kg. Hence, the certified ports benefitted from a price premium of 15.2% over the average price of the uncertified product.	The average price per kg has doubled between 2001 and 2021 from Euro 3.87 to Euro 8.15				
	Opportunity for alternative livelihoods	Artisanal fishermen are highly dependent on fishing activities. For most ship-owners, fishing constitutes their only source of income.	Galicia's coastal territory can be considered as fishing-dependent. Five of the nine Maritime Zones are highly dependent; three are moderately dependent and only one has low dependency. Zones with				

Rapfish evaluation		Common octopus value chain (Asturias and Galicia)					
**Sustainability indicators**	**Attributes**	**Producers**		**Intermediaries (Collection from 1st producer point)**	**Processors (Preparation of value-added products)**	**Distributors (Distribution/logistics)**	**Retailers (Wholesalers, HORECA, fishmongers, supermarkets)**
		**Asturias**	**Galicia**				
			intermediate population densities belong to the group of zones that are more heavily dependent on fishing. Nevertheless, the level of dependency is lower in urban zones, suggesting opportunities for alternative employment exist in these zones.<br><br>The small-scale fisheries segment generates a greater volume of employment than income, whereas for the coastal fleet the opposite is more often the case. Therefore if operational objectives of ecosystem management consist of increasing or protecting employment in the Galician aquaculture and fisheries sector, the measures ought to be focused on intervention and aid for the more artisanal segments. If the priority aims consist of generating income, the measures adopted ought to be aimed at the more industrialized sectors with higher average outputs.				
	Marketing system	Prior to certification, most of the western octopus fleet boats chose to sell their catches in the auctions of their own home ports. The main buyers were wholesalers, although a small part of the sales in the auctions were made directly to retailers and restaurants. There was a high degree of concentration and buyer power.<br><br>Market research was conducted on the eco-labelled octopus in Central Europe: Italy, France and the Benelux. The study analyzed the possibilities of accessing to the	An analysis of the relationship between the auction and wholesale links of the Galician common octopus markets confirmed that the average price of octopus was much higher at the wholesale level than the auction level and that variations in the price in any of the two levels of the marketing chain considered would lead to variations in the price of the other link, thus not confirming the leadership of one market over another. However, the causal relationship between prices at the auction level and prices at the	In Galicia there is an open harbour auction system in 36 fish markets around the coast. The highest quantities (I Jan 2021 – 4 Nov 2021) were sold in Ribeira (235,386 kg), A Coruna (total 225,583 kg) and Vigo (129,220). Sales prices differ at each market with highest average sales prices for this period found in Redondela (11.61 Euros/kg), Barallobre (10 Euros/kg) and Cangas (9 Euros/kg).			

Rapfish evaluation		Common octopus value chain (Asturias and Galicia)					
**Sustainability indicators**	**Attributes**	**Producers**		**Intermediaries (Collection from 1st producer point)**	**Processors (Preparation of value-added products)**	**Distributors (Distribution/logistics)**	**Retailers (Wholesalers, HORECA, fishmongers, supermarkets)**
		**Asturias**	**Galicia**				
		international market. A commercial plan for the certified octopus was elaborated. MSC chain of custody certified traders were contacted. Commercialization tests were carried out. A sale system was established, which consists of a future auction (auction of the captures to be made in the next fifteen days). The first total campaign with certified octopus was 2016-2017.<br><br>Certification facilitated access to new markets which led to an increase in and diversification of demand.<br><br>Most production (around 80 tons every year) is sold to a Spanish firm specializing in the transformation of octopus which sells the processed product with the MSC label abroad (e.g. the USA, Canada, Japan).	wholesale level is stronger than the relationship in the opposite direction. In short, the existence of a stable long-term connection between both links in the octopus marketing chain reveals the possibility of implementing measures that reinforce market power and, therefore, the position of the fishermen in the pricing mechanism. However, the process of fixing prices in markets through public auctions does not allow important characteristics of the Galician product (e.g. quality, freshness) to be sufficiently highlighted. To strengthen the power of the fishermen, management measures should be implemented aimed at adding value to the product at origin (thus allowing the increase in the income level of the fishermen), or else, in another management, favoring a change in the business dimension or the system of fishing rights, in such a way that they allow a broader margin of strategic action by companies. The regulation must make fishing activity compatible, in any case, with the conservation of populations and the balance of ecosystems in the medium and long term. All these considerations may be of special interest due to the importance of the contribution of fishing exploitation for Galicia.	Auctions sell landings from local fleets and take place at harbours. Traditionally, local wholesalers, retailers and restaurateurs were the main buyers at the auctions, but today large wholesalers and retail chains are also present, mostly at all important auctions along the coast.			

Rapfish evaluation		Common octopus value chain (Asturias and Galicia)					
Sustainability indicators	Attributes	Producers		Intermediaries (Collection from 1st producer point)	Processors (Preparation of value-added products)	Distributors (Distribution/logistics)	Retailers (Wholesalers, HORECA, fishmongers, supermarkets)
		Asturias	Galicia				
	Equity of economic benefits	There is no clear hierarchy among crewmembers and the retribution system is by shares of the net profits.<br><br>Evidence of a price premium for certified octopus products is very important because most former studies about the price premium in eco-labelled seafood products were based mainly on consumer preferences and they did not reflect clearly whether this premium was also obtained in other stages of the value chain.		First sale price for fresh octopus landed in Galicia includes auction fees and may be half the final retail price.<br><br>First sale price in Galicia has increased from 6 Euros/kg (2011) to 9 Euros/kg (2021), however quantities have dropped from 3.4 million kg (2011) to 1.3 million kg (2021).<br><br>Ports of Ribeira, A Coruna and Vigo sold 44% of fresh octopus sold in Galicia in 2020.	Costs for the processor to transform fresh Galician octopus for sale frozen in a supermarket include gutting losses, processing costs (freezing, packaging, transport) and processor margin.<br><br>In 2017, Frigoríficos Rosa de los Vientos, S.L. was a finalist in the Blue Economy Awards and created 4 new jobs in collaboration with Emprega Marín, Cogami and the Red Cross, thanks to the introduction of Octopus Fumet, its new production line.<br><br>Cost of defrosting, gutting, tenderizing, cooking losses, other operational costs (transport, packaging, energy, labour).	Transport costs.	Retail price can be double the first sale price due to wholesaler costs, purchase price for retail platform, transport to retailer, retailer costs and margin and VAT.
	Commoditizatio n	Another important finding of this research is that the positive impact of eco-labelling and certification on O. vulgaris prices is the time span necessary to get the economic benefits since the observed effect has been obtained after the first year of the MSC implementation. One explanation of this result could be in that MSC-certified octopus is a product with a very limited supply (around 80 tons in Spain and 190 tons in Australia), but an increasing demand in the world. Hence, considering the economic benefit obtained by fishers and the speed to	There is a desire among some fishers to develop marketing around an easy to identify product, with consistent quality at the regional level, to encourage consumers to choose it as a local resource from an environmentally sustainable artisanal fishery.		The Xunta de Galicia has two certification programs providing hallmarks of quality and provenance for the promotion and defence of Galician products. 'Galicia Calidade' (https://www.galiciacalidade.gal/ES/home) certifies the origin of Galician products and 'pescadeRias' (https://deondesenon.xunta.gal/es) certifies products have been fished by the artisanal fleet in Galicia.		Pulponor is the authorised distributor of the 'Polbo de Lonxa' brand under which the octopus caught by the cofradias from Finisterre, Corcubion, Lira, Murros, Ou Pindo and Porto de Son is sold through the virtual platform 'Lonxa na Rede'. Pulponor's 'Polbo de Lonxa' brand guarantees the origin and quality of the octopus to consumers. The 'Polbo de Lonxa' label identifies the product at point of sale and in

Rapfish evaluation		Common octopus value chain (Asturias and Galicia)					
**Sustainability indicators**	**Attributes**	**Producers**		**Intermediaries (Collection from 1st producer point)**	**Processors (Preparation of value-added products)**	**Distributors (Distribution/logistics)**	**Retailers (Wholesalers, HORECA, fishmongers, supermarkets)**
		**Asturias**	**Galicia**				
		obtain it, we can infer that eco-labelling is a good tool to differentiate seafood products in the market, especially for small or artisanal productions.			Some octopus processing companies have been certified 'Galicia Calidade', (e.g. Gallego Pereira, Rosa de los Vientos), indicating the raw materials are of Galician origin or provide added value.<br><br>Frigorifico Moldes octopus products are certified 'pescadeRias' signifying they come from inshore fishing that is landed daily in the ports of the Autonomous Community of Galicia.<br><br>Frigoríficos Rosa de los Vientos, S.L. has established a new line including the revaluation of by-products, which allows it to market a new product: "Octopus croquettes". With this investment with funding from the European Union, Frigoríficos Rosa de los Vientos, S.L. contributes to the valorization and promotion of local fishing products, favoring employment, social inclusion and economic growth of the territory.		the hospitality establishments where it is sold.<br><br>The Lonxa na Rede platform was born with the aim of making the 'Polbo de Lonxa' brand accessible to large and medium-sized buyers through the network without them having to physically go to the market. This way they will be able to offer their customers 'Polbo de Lonxa': an octopus with its own Galician brand of quality and good price.<br><br>Asturpesca has been certified under the MSC Chain of Custody standard since 2016 in favour of sustainable fishing which covers distribution of common octopus caught with traps by boats belonging to the Tapia de Casariego, Viavelez, Puerta de Vega and Ortiguera fishermen's guilds (cofradias).
	Import/export markets	Prior to MSC certification, the marketing of octopus caught within the Management Plan took place mainly through the Asturian fish markets, with a relatively low percentage being sold outside of them.	Galicia has a centuries long history of exporting octopus products to other Spanish regions and overseas (e.g. Portugal).	Price of frozen octopus imported into Galicia from Morocco and sold cooked by a Spanish retailer may be one third of the retail price.			

Rapfish evaluation		Common octopus value chain (Asturias and Galicia)					
**Sustainability indicators**	**Attributes**	**Producers**		**Intermediaries (Collection from 1st producer point)**	**Processors (Preparation of value-added products)**	**Distributors (Distribution/logistics)**	**Retailers (Wholesalers, HORECA, fishmongers, supermarkets)**
		**Asturias**	**Galicia**				
		Currently 90% of the certified catch is destined for export.<br><br>Most eco-labelled production (around 80 tons every year) is sold to a Spanish firm specializing in the transformation of octopus which sells the processed product with the MSC label abroad (e.g. the USA, Canada, Japan).<br><br>Small amounts of MSC certified Asturias octopus (15 tonnes/year) are directly exported via traders to markets where the demand for eco-labelled fish products is high (Northern Europe, USA, and Switzerland). The price premium usually reaches +20-25%.					
	Value transmission	Before MSC certification, the octopus' price in all ports of Asturias was very similar with an average of the annual price around 4.89 €/Kg. After 2015, there was a general increase in market prices of Asturian octopus, although this increase has been more relevant for those ports with the MSC certification. Thus, the average of the annual prices in MSC-certified ports increased up to 8.01 €/Kg versus the average of the rest of the ports in Asturias with an average price of 6.93 €/Kg. Hence, the certified ports benefitted from a price premium of 15.2% over the average price of the uncertified product.					The Pulponor 'Polbo de Lonxa' label identifies at point of sale and in the hospitality establishments where it is sold that the octopus product is caught by the cofradias from Finisterre, Corcubion, Lira, Murros, Ou Pindo and Porto de Son. The brand guarantees the origin and quality of the octopus to consumers<br><br>The Pulponor Lonxa na Rede platform makes the 'Polbo de Lonxa' brand accessible to large and medium-sized buyers through the network without them having to physically go to the market. This way they will be able to offer their customers 'Polbo de Lonxa': an octopus

Rapfish evaluation		Common octopus value chain (Asturias and Galicia)					
**Sustainability indicators**	**Attributes**	**Producers**		**Intermediaries (Collection from 1st producer point)**	**Processors (Preparation of value-added products)**	**Distributors (Distribution/logistics)**	**Retailers (Wholesalers, HORECA, fishmongers, supermarkets)**
		**Asturias**	**Galicia**				
							with its own Galician brand of quality and good price.<br><br>The Asturpesca 'Pulpo MSC Fresco' label on its octopus products transmits the value of the product by guaranteeing to the consumer that the product is traceable and originated from identifiable sustainably managed fisheries.
Social evaluation	Strength of social network	The Octopus Management Plan is a co-management tool for the species of interest. In this regard, local fishing guilds (located in the ports of Cudillero, Oviñana, Luarca, Puerto de Vega, Ortiguera, Viavélez,Tapia de Casariego and Figueras) and regional authorities cooperate to define regulatory measures and monitor key parameters in the octopus fishery. Each season, small-scale boats officially registered as *multi-gear in the Cantabrian and Northwest fishing ground – CNW* (and, exceptionally, some bottom long-liners) based at the aforementioned eight fishing ports apply to the regional fishing authority for a license to operate in this fishery.<br><br>As a result of the ecolabelling process, members of different fishermen guilds (cofradias) work together for the first time and create the association "ARPESOS" (Association of Shipowners of the Octopus Fisheries with a Sustainability Certificate).					Pulponor is a cooperative that groups orders and services in such a way that their customers obtain significant savings in price and cost. They act as a purchasing centre to establish strategic alliances for all parties to obtain benefits.<br><br>Pulponor claim the following principles and values of the International Cooperative Alliance: commitment to the community; promotion of training, information and education; voluntary membership; autonomy and independence; cooperation between cooperatives; democratic management; economic participation.

Rapfish evaluation		Common octopus value chain (Asturias and Galicia)					
**Sustainability indicators**	**Attributes**	**Producers**		**Intermediaries (Collection from 1st producer point)**	**Processors (Preparation of value-added products)**	**Distributors (Distribution/logistics)**	**Retailers (Wholesalers, HORECA, fishmongers, supermarkets)**
		**Asturias**	**Galicia**				
		Cofradias offer social assistance to fisher members, manage fishing activities inside their coastal territories of influence, have power over membership and fishing rights, integrate either the whole population or those belonging to a specific fishery in a fishing town, and include both vessel owners and crew.					
	Leadership	High media impact of the project enables the fishery to be identified as the first MSC-certified octopus worldwide with the Navia-Porcía region. Fishermen effort in favor of sustainability is recognized.					Pulponor states the following values on its website: mutual help, responsibility, democracy, equality, equity, solidarity.<br><br>Pulponor claim the following principles and values of the International Cooperative Alliance: commitment to the community; promotion of training, information and education; voluntary membership; autonomy and independence; cooperation between cooperatives; democratic management; economic participation.<br><br>Asturpesca claims social responsibility by committing to improving their socio-economic environment and rejecting behaviours that violate the dignity of people. Asturpesca claims to firmly believe in equal opportunities and non-discrimination for reasons of age, gender or country of origin, resulting in a workforce in which diversity is one of its values. Women, account for more than 60% of the workforce, and lead the majority of positions of responsibility in the company
		Individual cofradias are organised into regional and national federations to promote the interests of fishing members and communities.					

Rapfish evaluation		Common octopus value chain (Asturias and Galicia)					
**Sustainability indicators**	**Attributes**	**Producers**		**Intermediaries (Collection from 1st producer point)**	**Processors (Preparation of value-added products)**	**Distributors (Distribution/logistics)**	**Retailers (Wholesalers, HORECA, fishmongers, supermarkets)**
		**Asturias**	**Galicia**				
							and the Management Committee is mostly female.
	Extent of Local Environmental Knowledge [LEK]	Artisanal fishing knowledge has traditionally been transmitted from one generation to another.	Fishers contribute LEK to on-board observations (e.g. seafloor type) and modification of annual experimental management plans according to the particular topography of the coast and the status of the resource.<br>Some fishers were involved in mapping the octopus fishing grounds based on their traditional ecological knowledge (TEK)				
	Fishers to owners ratio	95.5% of the ship-owners work on board as one more crewmember. Artisanal vessels are normally family owned where there is no clear hierarchy among crewmembers and the retribution system is by shares of the incomes.<br>1-3 crewmembers per vessel.	Mainly family companies. There is an average of 2-3 crewmembers per vessel (range 1-6), suggesting 2400-3600 fishers are involved in the fishery.				
Ethical evaluation	Adjacency	Most artisanal crewmembers come from coastal communities close to the fishing port where the vessels operate confirming the existence of strong bonds between the artisanal fleet and regional coastal communities.	Octopus is caught in intertidal and subtidal zones close to the coastline and auctioned at 51 fish markets around the coast.<br>Fleets operate in the Rias with mixed gears, catching high value species (e.g. crustaceans, mollusks, ground fish) which are sold fresh in the fish markets of each maritime zone at daily auctions.<br><br>Cofradias' positive values include: a) proximity to and relationship with the environment, with a high level of knowledge and dependency; b) signs of identity, linked to the population's identification with the sea and the marine environment; c) associative tradition, including the culture of collaboration and adaptive ability, also of a strategic nature [8].				Pulponor is situated in Santiago de Compostela, Galicia, in close proximity to the artisanal fisheries that supply their octopus products.<br><br>Asturpesca is situated in Aviles, Asturias, in close proximity to the artisanal fisheries that supply their octopus products.

**Rapfish evaluation**		**Common octopus value chain (Asturias and Galicia)**					
**Sustainability indicators**	**Attributes**	**Producers**		**Intermediaries (Collection from 1st producer point)**	**Processors (Preparation of value-added products)**	**Distributors (Distribution/logistics)**	**Retailers (Wholesalers, HORECA, fishmongers, supermarkets)**
		**Asturias**	**Galicia**				
	Iconicity	Regional icon: O. vulgaris is one of the main seven species fished by artisanal fleets.	Regional icon: O. vulgaris has been fished in Galicia since at least the 12th Century AD and is one of the most important marine invertebrates fished in the region.<br>Consumption of octopus at traditional popular fairs is currently a preeminent activity				Pulponor's 'Polbo de Lonxa' brand guarantees the origin and quality of the octopus to consumers. The 'Polbo de Lonxa' label identifies the product at point of sale and in the hospitality establishments where it is sold.
	Equity of access	Current generational replacement perspectives for the artisanal vessels are quite low in terms of ship-owners passing on the business to younger members of the family. The longer the vessel the higher the probability of a younger family member taking on the activity in the future possibly because longer vessels are less dependent on climatic conditions therefore longer vessels are able to generate a more stable income flow, which is more attractive to younger generations. The lack of generational replacement is one of the main barriers to achieving desired long-term sustainability. This is aggravated by excessive formative exigencies and administrative constraints to recruit new and young fishermen.<br><br>According to the Octopus Management Plan, the fishery remains open seven months a year (from 15 December to 15 July), when vessels are allowed to fish octopus under request to the fishing authority. The fishery is operated mainly by small-scale multi-gear boats that catch octopus.	Local government regulatory measures include: a fishing permit/license (maximum 5 gears per vessel), with restrictions on authorised fishing activities (e.g. fishing times, zones, catch limits, number of traps according to vessel size/number of fishers, gear size, identification chips).				

Rapfish evaluation		Common octopus value chain (Asturias and Galicia)					
**Sustainability indicators**	**Attributes**	**Producers**		**Intermediaries (Collection from 1st producer point)**	**Processors (Preparation of value-added products)**	**Distributors (Distribution/logistics)**	**Retailers (Wholesalers, HORECA, fishmongers, supermarkets)**
		**Asturias**	**Galicia**				
		Only artisanal fleets are authorised to fish for octopus.					
	Just governance	The Octopus Management Plan is a co-management tool by which local fishing guilds (located in the ports of Cudillero, Oviñana, Luarca, Puerto de Vega, Ortiguera, Viavélez,Tapia de Casariego and Figueras) and regional authorities cooperate to define regulatory measures and monitor key parameters in the octopus fishery.	There are three management zones in the Galicia region (A, B, C) and three different co-management plans (one per zone). Annual experimental management plans are implemented differently along the coast, and allow modifications or complementing of the general basic rules according to the particular topography of the coast and the status of the resource in co-management with the local fishers				
	Illegal, unreported, and unregulated (IUU) fishing		There is a substantial amount of illegal commercial catch, which has been estimated to range between 20–50 % of the total reported catches in 2010, and there is also a substantial number of recreational fishers selling octopus directly to restaurants, illegally. There was a discrepancy of 30 % in catches between 1998 and 2000, which can be due to misreporting or illegal selling (out the official auctions). However, better control and monitoring programs appear to have contributed to a substantial reduction of illegal practices over the last few years. In addition, advances in co-management processes in recent years have reduced social conflicts, and consequently increased compliance with regulations.<br><br>There was a notable mismatch between estimated catches and official landings that decreased towards the final part of the analysed				

Rapfish evaluation		Common octopus value chain (Asturias and Galicia)					
**Sustainability indicators**	**Attributes**	**Producers**		**Intermediaries (Collection from 1st producer point)**	**Processors (Preparation of value-added products)**	**Distributors (Distribution/logistics)**	**Retailers (Wholesalers, HORECA, fishmongers, supermarkets)**
		**Asturias**	**Galicia**				
			period (2001 – 2016), suggesting a certain level of misreporting.<br><br>Fisheries experts suggest re-evaluating the number of traps per vessel and fisher to obtain a decent income as in some cases the current legal number is so low that some fishers are operating with illegally high numbers of traps.				
	Mitigation of harm		Non-target species (e.g. invertebrates) are discarded				
	Consumer/buyer choice	MSC certified octopus is included in the brand "Alimentos del Paraíso“ (Food of Paradise).	The Xunta de Galicia has two certification programs providing hallmarks of quality and provenance for the promotion and defence of Galician products. ‘Galicia Calidade’ (https://www.galiciacalidade.gal/ES/home) certifies the origin of Galician products and ‘pescadeRias’ (https://deondesenon.xunta.gal/es) certifies products have been fished by the artisanal fleet in Galicia.		Some octopus processing companies have been certified ‘Galicia Calidade’, (e.g. Gallego Pereira, Rosa de los Vientos), indicating the raw materials are of Galician origin or provide added value.<br><br>Frigorifico Moldes octopus products are certified ‘pescadeRias’ signifying they come from inshore fishing that is landed daily in the ports of the Autonomous Community of Galicia.<br><br>Frigoríficos Rosa de los Vientos, S.L. has established a new line including the revaluation of by-products, which allows it to market a new product: "Octopus croquettes". With this investment with funding from the European Union, Frigoríficos Rosa de los Vientos, S.L. contributes to the valorization and		Pulponor’s ‘Polbo de Lonxa’ brand guarantees the origin and quality of the octopus to consumers. The ‘Polbo de Lonxa’ label identifies the product at point of sale and in the hospitality establishments where it is sold.<br><br>Asturpesca, as part of the MSC Chain of Custody requirement, provides the following kinds of information on its octopus product labels: common and scientific species names, FAO code, capture zone, fishing gear, boat identification number, port of disembarkation, date of capture, 1st buyer/supplier, lot number.<br><br>In addition, their website hosts the MSC CoC certificate which can be downloaded and which contains the following information about their octopus product: quality specifications; biological,

**Rapfish evaluation**		**Common octopus value chain (Asturias and Galicia)**					
**Sustainability indicators**	**Attributes**	**Producers**		**Intermediaries (Collection from 1st producer point)**	**Processors (Preparation of value-added products)**	**Distributors (Distribution/logistics)**	**Retailers (Wholesalers, HORECA, fishmongers, supermarkets)**
		**Asturias**	**Galicia**				
					promotion of local fishing products, favoring employment, social inclusion and economic growth of the territory.		physical and chemical characteristics; labelling information; expected use; storage conditions; product shelf-life; and associated regulations.
	Traceability	MSC chain of custody certified traders were contacted.	Octopus caught in the Lugo province fishery is coded with a vessel identification bar at the landing site and that identification continues until auction.<br><br>Polbo das Rías is the first collective<br>from its fishing. Behind Polbo das R<br>extraction of octopus in the rich Ga<br>alternatives to differentiate and ensur<br>The Polbo das Rías Collective Brand<br>1. A quality control of the octopus from its capture (laboratory control of hygiene parameters).<br>2. Origin of the octopus caught in the waters of the Galician Rías Baixas.<br>3. Origin of the Galician artisanal fleet.<br>4. Individual identification of each octopus: individual traceability.<br>5. Origin exclusively from extractive fishing.	Gallego Pereira and Frigorificos Moldes claim on their websites to purchase fresh octopus directly from the Galician fish markets.	Rosa de los Vientos and Frigorificos Moldes claim on their websites to process octopus from the Rias Gallegas.<br><br>Fesba claims on its website to process octopus from FAO 27 (Spain and Portugal). They also claim 'Rigorous traceability control and strict compliance with current National and European consumer information regulations'		Froiz supermarket chain provides product characteristics on its online shopping website including scientific name of species, method of production, fishing gear used, capture zone and quality.<br><br>Gadis only provides scientific name for its cooked O. vulgaris products – brand name 'el Rey del Pulpo'<br><br>Pulponor is the authorised distributor of the 'Polbo de Lonxa' brand under which the octopus caught by the cofradias from Finisterre, Corcubion, Lira, Murros, Ou Pindo and Porto de Son is sold through the virtual platform 'Lonxa na Rede'.<br><br>Pulponor's 'Polbo de Lonxa' brand guarantees the origin and quality of the octopus to consumers. The 'Polbo de Lonxa' label identifies the product at point of sale and in the hospitality establishments where it is sold.<br><br>Asturpesca, as part of the MSC Chain of Custody requirement,

Rapfish evaluation		Common octopus value chain (Asturias and Galicia)					
**Sustainability indicators**	**Attributes**	**Producers**		**Intermediaries (Collection from 1st producer point)**	**Processors (Preparation of value-added products)**	**Distributors (Distribution/logistics)**	**Retailers (Wholesalers, HORECA, fishmongers, supermarkets)**
		**Asturias**	**Galicia**				
							provides the following kinds of information on its octopus product labels: common and scientific species names, FAO code, capture zone, fishing gear, boat identification number, port of disembarkation, date of capture, 1st buyer/supplier, lot number.<br><br>In addition, the Asturpesca website hosts the MSC CoC certificate which can be downloaded and which contains the following information about their octopus product: quality specifications; biological, physical and chemical characteristics; labelling information; expected use; storage conditions; product shelf-life; and associated regulations.
Institutional evaluation	Legitimacy	There has been a clear improvement of the reputation of the fishery in the region. They have obtained or at least reinforced the "Social Licence" that allows them to operate in their places. Since obtaining the MSC certification the fishery took part and appeared in many interviews in radio, TV, social media, press, etc and reinforced the perception of their activity as legitimate and socially, economically and environmentally sustainable.	Fisheries management in this region is shared by two administrations, the Galician Autonomous Government (Xunta de Galicia) and the National Government. The former is responsible for the management, monitoring and control of fisheries in Galician inshore waters, while the latter regulates offshore waters. There is a specific legal corpus for fisheries in Galician inshore waters with several regulations in force for the octopus fishery, setting rules about the gear which can be deployed, operating procedures, area of operation for different types				Pulponor claim the following principles and values of the International Cooperative Alliance: commitment to the community; promotion of training, information and education; voluntary membership; autonomy and independence; cooperation between cooperatives; democratic management; economic participation.

Rapfish evaluation		Common octopus value chain (Asturias and Galicia)					
**Sustainability indicators**	**Attributes**	**Producers**		**Intermediaries (Collection from 1st producer point)**	**Processors (Preparation of value-added products)**	**Distributors (Distribution/logistics)**	**Retailers (Wholesalers, HORECA, fishmongers, supermarkets)**
		**Asturias**	**Galicia**				
			of octopus traps, maximum amount of traps per vessel, the minimum landing weight, and the annual management plans for the octopus fishery. The management plan is established by fishing season (from June to May of the following year) and is usually implemented differently along the coast, allowing for modifications or complementing the general basic rules, according to the status of the resource, in co-management with the local fishers. The most recent management plan established the rules for the octopus fishery for the 2020–2021 season, and includes, amongst other measures, closed seasons (from May 29th to July 1st, 2020), minimum weight of catches (currently 1 kg), maximum daily catches taking into account the number of crew members onboard of the vessel, and the number of traps per working hour at sea.<br><br>The Xunta de Galicia has fisheries management powers in internal waters (e.g. maritime areas between the rias and the coastline), therefore the octopus fishing sector depends on the definition of rights, planning and regulation by the Xunta which has developed its own fisheries Laws: enshrined in Law 11/2008, modified by Law 6/ 2009, including operational decrees and directives.<br><br>Galicia has 63 cofradias with approximately 12,700 members. The cofradias are the most				

Rapfish evaluation		Common octopus value chain (Asturias and Galicia)					
**Sustainability indicators**	**Attributes**	**Producers**		**Intermediaries (Collection from 1st producer point)**	**Processors (Preparation of value-added products)**	**Distributors (Distribution/logistics)**	**Retailers (Wholesalers, HORECA, fishmongers, supermarkets)**
		**Asturias**	**Galicia**				
			traditional figure in terms of fishers adopting means of association via collective action strategies with regard to production, commercialization and representation processes, or in response to exercising certain collectively defined rights. Cofradias are regulated by Law 9/1993 (with modifications) and Decree 8/2014 which develop regulations at constitutional, collective action and operational levels.				
	Transparency	Pre-assessment of the fishery was conducted according to MSC standards with the aim of detecting weak points which were corrected before starting the certification process.<br><br>The evaluation of the fishery was carried out and medium-term improvement actions for sustainability were planned. External auditors assessed compliance with 3 principles and 28 indicators of sustainability. After more than a year of evaluation, on February 10th, 2016, Navia-Porcía fishing fleet obtained the first M.S.C. certification for an octopus fishery worldwide. As part of the monitoring phase, an action plan was designed to improve 4 sustainability indicators with a lower score. This plan included actions for the period 2017-2021. Annual MSC evaluation 2017: The actions of the improvement plan were conducted in the first year and	Cofradias in Galicia exhibit several weaknesses including: a) limits in the determination of the scope of actions and the definition of aims: limits as a business; uncertainty as to the rules and possibilities for the future; b) limited control over and ability to influence market processes and globalisation; c) limited influence over the future of the regulatory framework; d) confluence or interference of individual interests exercised in continuous short-term actions, with collective interests and a long-term vision of sustainability: this confluence generally stems from situations where abilities to define strategies and agreements are restricted (high transaction costs). In this context, the tendency to replicate conservative (not conservationist) positions can be better understood, opening the door to individual actions with opportunistic, short-term objectives.				Asturpesca, as part of the MSC Chain of Custody requirement, provides the following kinds of information on its octopus product labels: common and scientific species names, FAO code, capture zone, fishing gear, boat identification number, port of disembarkation, date of capture, 1st buyer/supplier, lot number.<br><br>In addition, their website hosts the MSC CoC certificate which can be downloaded and which contains the following information about their octopus product: quality specifications; biological, physical and chemical characteristics; labelling information; expected use; storage conditions; product shelf-life; and associated regulations.

Rapfish evaluation		Common octopus value chain (Asturias and Galicia)					
Sustainability indicators	Attributes	Producers		Intermediaries (Collection from 1st producer point)	Processors (Preparation of value-added products)	Distributors (Distribution/logistics)	Retailers (Wholesalers, HORECA, fishmongers, supermarkets)
		Asturias	Galicia				
		the results of the first external audit were positive therefore the certification of the fishery is valid to October 2021.<br><br>The management plan is regulated annually by means of a Resolution that is published in the Official Gazette of the Region (named B.O.P.A.).					Additional information on the website about the octopus product includes: capture zone, production method, presentation, packaging, ingredients.
	Accountability	The fishery is regularly monitored according to the MSC Standard.	Cofradias in Galicia:<br>Constitutional level. Legislation at State and Autonomous Community level grants cofradías and the fishermen affiliated to them certain rights. This is referring both to resource extraction rights as well as to those relating to dialogue and collaboration for management purposes. Changes do not occur easily in the short term and, in any case, would be determined in a context of interests and multiple representation (parliaments) with, therefore, minimum impact on the part of cofradías and fishermen.<br><br>Level of collective action. On this level, action plans are developed and the general rights established on the previous level are developed. Cofradías and fishermen participate and are consulted in this process. But the final decision corresponds to the Administration, in this case, fundamentally to the Autonomous Community Administration. This is why objectives and regulation criteria are the responsibility of the Public Administrations. Without entering into an evaluation of past				Asturpesca, is certified under the MSC Chain of Custody standard in favour of sustainable fishing, which covers distribution of their octopus products and is valid from 08/04/2022 to 07/04/2025.

**Rapfish evaluation**		**Common octopus value chain (Asturias and Galicia)**					
**Sustainability indicators**	**Attributes**	**Producers**		**Intermediaries (Collection from 1st producer point)**	**Processors (Preparation of value-added products)**	**Distributors (Distribution/logistics)**	**Retailers (Wholesalers, HORECA, fishmongers, supermarkets)**
		**Asturias**	**Galicia**				
			phases of processes, the general trend is and has been to maintain the status quo, proposing classic and short-range adjustments in the face of problems (basic modifications via inputs, outputs and closures), trying to "resolve complaints" by minimising changes and impacts and not declaring medium or long-term objectives.<br><br>Operational level. This is the level where there is room for individual initiatives on the part of cofradías in management affairs. Here, the aforementioned confluence or interference of collective and individual interests is perceived. To the extent that the room for manoeuvre is limited and the participants multiple, agreements on maximums are improbable and opportunistic actions increase. This trend augments if i) there are disincentives for compliance: inadequate or inefficient control and penalty mechanisms; ii) there is a high degree of heterogeneity within each cofradía and between different cofradías.				
	Inclusiveness	Fishermen get involved in the management of the fishery through the "Consultative Committee" by taking part in decision making.	The management plans for the octopus fisheries involved the public administration, the fishing sector, NGOs, and scientists.<br><br>However, an important conflict was identified as approval by the Autonomous Government of Galicia of an Annual Management plan for coastal octopus without the				Asturpesca claims social responsibility by committing to improving their socio-economic environment and rejecting behaviours that violate the dignity of people. Asturpesca claims to firmly believe in equal opportunities and non-discrimination for reasons of age, gender or country of origin., resulting in

Rapfish evaluation		Common octopus value chain (Asturias and Galicia)					
**Sustainability indicators**	**Attributes**	**Producers**		**Intermediaries (Collection from 1st producer point)**	**Processors (Preparation of value-added products)**	**Distributors (Distribution/logistics)**	**Retailers (Wholesalers, HORECA, fishmongers, supermarkets)**
		**Asturias**	**Galicia**				
			unanimous approval of the Galician artisanal sector.<br><br>Historically, fishers had barely participated in octopus fishing decision-making until the implementation of a Monitoring Committee of the Annual Octopus Management Plan in 2013, made up of representatives of the fishing sector and of the Autonomous Fisheries Government, included in the current annual regulation of this fishery.<br><br>Belonging to the cofradia group in Galicia is linked to the territory and the activity, but now with the established legal requirements relating to the control of fishing intensity. The right to belong is defined by law, and the cofradía itself cannot exclude (which can, however, occur in other associations), but membership is non-obligatory. The group will, in any case, adopt it as a protection system for its own members against outsiders.				a workforce in which diversity is one of its values. Women, account for more than 60% of the workforce, and lead the majority of positions of responsibility in the company and the Management Committee is mostly female.
				Other professions linked to the sea are no longer a part of the small-scale fishing communities and are not taken into account in the current legal make-up of the cofradías, such as commercial activities beyond the first sale, supplies, processing, equipment and the construction of fishing vessels, etc. In fact, under the current legal definition, the consultation, collaboration and representation functions of cofradías refer expressly to the fisheries sector and those working in the industry.			
	Fairness	The Western Asturias Octopus Monitoring Committee facilitates engagement between fishermen, administration, scientists and NGOs to discuss management measures before they are formalised. Since certification, the fishery has delivered various economic, social and environmental benefits including higher relative prices for eco-labelled products where the premium is obtained across the	The cofradías in Galicia continue maintaining their objectives regarding social welfare, representation and insertion in the social fabric of Galicia. In the process, new social values and trends are incorporated, the activity and increased professionalism of women in the sector being widely recognised, especially in the shellfishing sector. In general, the idea of the group and cooperation is				Pulponor states the following values on its website: mutual help, responsibility, democracy, equality, equity, solidarity.<br><br>Pulponor claim the following principles and values of the International Cooperative Alliance: commitment to the community; promotion of training, information and

Rapfish evaluation		Common octopus value chain (Asturias and Galicia)					
Sustainability indicators	Attributes	Producers		Intermediaries (Collection from 1st producer point)	Processors (Preparation of value-added products)	Distributors (Distribution/logistics)	Retailers (Wholesalers, HORECA, fishmongers, supermarkets)
		Asturias	Galicia				
		value chain, market differentiation, new markets, better governance (e.g. raising awareness among fishers about the positive role of science in fisheries management) and improved stock health.	maintained, but short-term views and action co-exist (based more on collective and inter-generational interests).				education; voluntary membership; autonomy and independence; cooperation between cooperatives; democratic management; economic participation.
	Connectivity [coordination, cooperation]	Cofradias have a strong presence in coastal matters as representatives of local fishers However, their capability to sway policies is complex, partially due to their lack of technical staff in most cases. Furthermore, coordinating their demands with those of the public administration to further their interests can be difficult (Alegret 2000), taking into account the possible conflicts among organisations and leaderships. No less important are the linkages that sometimes develop between cofradía leaders and political parties, creating clientelism. Financing cofradías activities depends partially on regional government funds, and this further contributes to the complexity of relationships.					Pulponor is a cooperative that groups orders and services in such a way that their customers obtain significant savings in price and cost. They act as a purchasing centre to establish strategic alliances for all parties to obtain benefits.<br><br>Pulponor claim the following principles and values of the International Cooperative Alliance: commitment to the community; promotion of training, information and education; voluntary membership; autonomy and independence; cooperation between cooperatives; democratic management; economic participation.
		Fishermen as well as managers and researchers became part of the certification process through meetings and technical conferences.<br><br>Cooperation among different local agents as fishers, fishers' guilds, and local authorities to implement the MSC eco-labelling schemes in the small-scale fishery provides economic and environmental benefits for coastal communities which depend on traditionally artisanal fishing methods.	The potential for cooperation is substantive but the attributes of the governance system and actors have hampered this possibility. If the experiences of cooperation in the past are an important factor in building trust, relationships of conflict or impunity in the face of non-compliance with the rules decrease trust and reduce the reciprocity of those willing to cooperate to maintain the resource.				

Rapfish evaluation		Common octopus value chain (Asturias and Galicia)					
Sustainability indicators	Attributes	Producers		Intermediaries (Collection from 1st producer point)	Processors (Preparation of value-added products)	Distributors (Distribution/logistics)	Retailers (Wholesalers, HORECA, fishmongers, supermarkets)
		Asturias	Galicia				
		Members of different fishermen guilds work together for the first time. Create the association "ARPESOS" (Association of Shipowners of the Octopus Fisheries with a Sustainability Certificate).	The diagnosis confirms a high level of anomie, linked to dysfunctions in the motivations of the actors to comply with the rules. Systematic non-compliance in turn discourages the formation of social norms, makes the violation of norms attractive rules given the low risk of being discovered, the low cost of the sanction or even the impunity for certain infractions. And, finally, those who would like to cooperate feel powerless and follow the dominant strategy. Failure of the governance system to incentivize and ensure cooperation reinforces the importance of trust. Lack of trust operates as a barrier to designing and maintaining management measures. Some fishermen may not comply with the formal rules because they believe or claim to know that others will not comply. In the octopus fishery in Galicia, the interaction between trust and institutions seems immersed in a perverse cycle in which the levels of cooperation are low and mistrust feeds on itself.				
	Conflict [resilience]		There are several potential levels of conflict, both within the Galician cofradías themselves as well as in relation with other agents of the same sector or the immediate environment (e.g. relating to management activities, information activities, information levels, decision-making processes, monitoring and control activities, and external relations activities). Staying with the former, in present-day cofradías the differences in				

Rapfish evaluation		Common octopus value chain (Asturias and Galicia)					
**Sustainability indicators**	**Attributes**	**Producers**		**Intermediaries (Collection from 1st producer point)**	**Processors (Preparation of value-added products)**	**Distributors (Distribution/logistics)**	**Retailers (Wholesalers, HORECA, fishmongers, supermarkets)**
		**Asturias**	**Galicia**				
			perceptions and interests are evident due to: i) questions of gender, with the significant incorporation of women; ii) the type of activity, fishing and shellfishing, and their different methods; iii) different ideological positions, in part fuelled by a partisan interest in controlling a public body.				
	Legality [compliance]	Spain is a signatory to UNCLOS and a member of WTO, UNCTAD and FAO agreements. Spain has established an Exclusive Economic Zone (EEZ) in the Atlantic Ocean (Law 15/1978). In 2017, Spain adopted the Royal Decree 363/2017 of 8 April establishing a framework for maritime spatial planning, which transposes into Spanish legislation Directive 2014/89/EC of the European Parliament and of the Council of 23 July 2015, establishing a framework for maritime spatial planning (MSP). The Ministry of Agriculture, Fisheries and Food is the responsible MSP authority for fisheries. The Law on the Protection of the Marine Environment (Ley de protección del medio marino, Law 41/2010) transposed into national legislation the Marine Strategy Framework Directive (MSFD) and regulates the maritime areas within the 'maritime-terrestrial public domain', which includes the territorial waters and natural resources in the exclusive economic zone and the continental shelf. Autonomous regions with jurisdiction over the marine environment (e.g. Asturias and Galicia) participate in all phases of the development and implementation of the Spanish Marine Strategies according to the Law on the Protection of the Marine Environment = MSFD).					
			The octopus fishery in Galicia is carried out according to management plans arranged by the regional government which include closed seasons (from May to July), minimum size of catches (currently 1 kg), maximum daily catches taking into account the number of crew members, limited number of traps of working hours at sea, and similar measures.<br><br>The recent decline in landings since 2010 is explained by the interaction				

**Rapfish evaluation**		**Common octopus value chain (Asturias and Galicia)**					
**Sustainability indicators**	**Attributes**	**Producers**		**Intermediaries (Collection from 1st producer point)**	**Processors (Preparation of value-added products)**	**Distributors (Distribution/logistics)**	**Retailers (Wholesalers, HORECA, fishmongers, supermarkets)**
		**Asturias**	**Galicia**				
			of environmental variations in the Galician estuaries (rias), pollution, overfishing, and ineffective monitoring of rules. Official catch figures are, however, offset by the illegal catch of Octopus vulgaris, which is estimated to range between 20–50% of total reported catches in 2010, while the number of recreational fishers selling octopus to restaurants is also substantial (Villasante et al. 2015). Nevertheless, better control and monitoring programs with more sustainable fishing appear to have contributed to a substantial reduction of illegal practices over the last years.				
	Effectiveness	The fishery is under the process of continuous improvement of the sustainability indicators. These are reviewed annually (annual external evaluation by independent auditors). New control measures of exploitation (HCR) which are sensitive to the evolution of the stock (e.g. quota per week and crew) and surveillance systems of fishing effort (e.g. marking off the traps) have been introduced.<br>The scientific campaigns have been intensified with the participation of the fishermen (GPs, data collection, etc.).					

References

Álvarez Ballesteros, Marta. 2018. "Gobernanza policéntrica en sistemas socio-ecológicos complejos: La gestión de la pesquería del pulpo común (Octopus vulgaris) en Galicia." PhD, Economía aplicada, Universidad de Vigo.

Amigo Dobaño, Lucy, Mª Dolores Garza Gil, and Manuel Varela Lafuente. 2009. "Transmisión de precios en la cadena comercial de la pesca costera gallega. El caso del pulpo." *Revista Española de Estudios Agrosociales y Pesqueros* 222:49-72.

Asturpesca. 2022. "Inicio." accessed 30/11/2022. https://asturpesca.com/.

Bañón, Rafael. 2014. "Historiografia del pulpo en Galicia." *Anuario Brigantino* 37:1-10.

Bañón, Rafael, Jaime Otero, José Manuel Campelos-Álvarez, Alberto Garazo, and Alexandre Alonso-Fernández. 2018. "The traditional small-scale octopus trap fishery off the Galician coast (Northeastern Atlantic): Historical notes and current fishery dynamics." *Fisheries Research* 206:115-128. doi: https://doi.org/10.1016/j.fishres.2018.05.005.

Bjorndal, Trond, Anna Child, and Audun Lem. 2014. Value chain dynamics and the small-scale sector: Policy recommendations for small-scale fisheries and aquaculture trade. In *FAO Fisheries and Aquaculture Technical Paper 581*. Rome, Italy: Food and Agriculture Organization of the United Nations.

Borges, Lisa, and Lucia Revenga. 2019. Lugo octopus trap fishery. FishFix.

Burch, Monica Veronesi, and Stephanie Maes. 2017. Boosting business along the fisheries value chain. edited by Directorate-General for Maritime Affairs and Fisheries European Commission, Director-General. Brussels, Belgium: FARNET.

Bureau Veritas. 2021. Western Asturias octopus traps fishery of artisanal cofradías: Public Certification Report. In *Marine Stewardship Council fisheries assessments*: Bureau Veritas.

European Commission. 2013. Vigo case study report. In *Studies for carrying out the Common Fisheries Policy: Lot 3 socio-economic dimensions in EU fisheries*: Directorate-General for Maritime Affairs and Fisheries.

European Commission. 2020. *Octopus in the EU : price structure in the supply chain : focus on Italy, Spain and Greece : case study*. Edited by Directorate-General for Maritime Affairs and Fisheries. https://data.europa.eu/doi/10.2771/633791: Publications Office.

European MSP Platform. 2020. Marine spatial planning country information profile - Spain. https://maritime-spatial-planning.ec.europa.eu/sites/default/files/download/spain_november_2020.pdf: EU Commission.

FARNET. 2018. MSC certification for the western Asturias octopus traps fishery. https://webgate.ec.europa.eu/fpfis/cms/farnet2/sites/default/files/8_resourcemanagementseminar-msc-octopus.pdf.

Fernández Sánchez, José L., José M. Fernández Polanco, and Ignacio Llorente García. 2020. "Evidence of price premium for MSC-certified products at fishers' level: The case of the artisanal fleet of common octopus from Asturias (Spain)." *Marine Policy* 119. doi: https://doi.org/10.1016/j.marpol.2020.104098.

Fernández-Rueda, Pino, and Lucía García-Flórez. 2007. "Octopus vulgaris (Mollusca: Cephalopoda) fishery management assessment in Asturias (north-west Spain)." *Fisheries Research* 83 (2):351-354. doi: https://doi.org/10.1016/j.fishres.2006.10.006.

Fesba. 2021. "Company." accessed 21/012/2021. https://www.fesba.net/en/company/.

Frigorifico Moldes. 2021. "Nosotros." Frigorifico Moldes, accessed 21/12/2021. https://www.frigorificosmoldes.com/somos-una-empresa-de-elaboracion-distribucion-y-venta-a-hosteleria-en-galicia/.

Froiz. 2021. "Pulpo." Froiz Supermercado, accessed 21/12/2021. https://www.froiz.com/mobile/4-4-1285-0-0/.

Gadisline. 2021. "Octopus products." Gadis, accessed 21/12/2021. https://www.gadisline.com/listado-de-productos/?hdSearch=pulpo.

Gallego Pereiro. 2021. "Inicio." Gallego Pereira, accessed 21/12/2021. http://gallegopereira.es/.

García-de-la-Fuente, Laura, Esteban Fernández-Vázquez, and Mª Carmen Ramos-Carvajal. 2020. "Sample selection bias in fisheries technical efficiency studies using stochastic frontiers; presence and correction for an artisanal fishery in Northwest Spain." *Ocean & Coastal Management* 198:105319. doi: https://doi.org/10.1016/j.ocecoaman.2020.105319.

García-de-la-Fuente, Laura, Javier González-Álvarez, Lucía García-Flórez, Pino Fernández-Rueda, and Jorge Alcázar-Álvarez. 2013. "Relevance of socioeconomic information for the sustainable management of artisanal fisheries in South Europe. A characterization study of the Asturian artisanal fleet (northern Spain)." *Ocean & Coastal Management* 86:61-71. doi: https://doi.org/10.1016/j.ocecoaman.2013.05.007.

García-Lorenzo, Iria, Manuel María Varela-Lafuente, and M. Dolores Garza-Gil. 2019. "Adaptative processes in small-scale traditional fishermen´s organisations. The case of Cofradías in Galicia (NW Spain)." *Marine Policy* 99:382-390. doi: https://doi.org/10.1016/j.marpol.2018.10.041.

González-Álvarez, Javier, Laura García-de-la-Fuente, Lucía García-Flórez, Mª del Pino Fernández-Rueda, and Jorge Luis Alcázar-Álvarez. 2016. "Identification and characterization of métiers in multi-species artisanal fisheries. A case study in northwest Spain." *Natural Resources* 07 (06):295-314. doi: https://doi.org/10.4236/nr.2016.76026.

Otero, Jaime, Xosé Antón Álvarez-Salgado, Ángel F. González, Ana Miranda, Steve B. Groom, José M. Cabanas, Gerardo Casas, Ben Wheatley, and Ángel Guerra. 2008. "Bottom-up control of common octopus Octopus vulgaris in the Galician upwelling system, northeast Atlantic Ocean." *Marine Ecology Progress Series* 362:181-192. doi: https://doi.org/10.3354/meps07437.

Otero, Jaime, Francisco Rocha, Ángel F. González, Joaquín Gracia, and Ángel Guerra. 2005. "Modeling artisanal coastal fisheries of Galicia (NW Spain) based on data obtained from fishers: the case of Octopus vulgaris." *Scientia Marina* 69 (4):577-585. doi: https://doi.org/10.3989/scimar.2005.69n4577.

Pascual-Fernández, José J., David Florido-del-Corral, Raquel De la Cruz-Modino, and Sebastián Villasante. 2020. "Small-Scale Fisheries in Spain: Diversity and Challenges." In *Small-Scale Fisheries in Europe: Status, Resilience and Governance*, edited by José J. Pascual-Fernández, Cristina Pita and Maarten Bavinck. Springer, Cham.

Pesca de Galicia. 2021. "Informes estatisticos." Xunta de Galicia, accessed 20/10/2021. https://www.pescadegalicia.gal/estadisticas.

Pesca de Galicia. 2022. "Guia de especies pesqueiras: Polbo." Xunta de Galicia Plataforma tecnolóxica da pesca, accessed 02/02/2022. https://www.pescadegalicia.gal/gl/guia-especies/detalle/OCC.

Pita, Cristina, Katina Roumbedakis, Teresa Fonseca, Fábio L. Matos, João Pereira, Sebastián Villasante, Pablo Pita, José Maria Bellido, Angel F. Gonzalez, Manuel García-Tasende, Evgenia Lefkaditou, Aggeliki Adamidou, Danila Cuccu, Paola Belcari, Ana Moreno, and Graham J. Pierce. 2021. "Fisheries for common octopus in Europe: socioeconomic importance and management." *Fisheries Research* 235:105820. doi: https://doi.org/10.1016/j.fishres.2020.105820.

Pita, Pablo, Duarte Fernández-Vidal, Javier García-Galdo, and Ramón Muíño. 2016. "The use of the traditional ecological knowledge of fishermen, cost-effective tools and participatory models in artisanal fisheries: Towards the co-management of common octopus in Galicia (NW Spain)." *Fisheries Research* 178:4-12. doi: https://doi.org/10.1016/j.fishres.2015.07.021.

Pitcher, T. J., M. E. Lam, C. Ainsworth, A. Martindale, K. Nakamura, R. I. Perry, and T. Ward. 2013. "Improvements to Rapfish: a rapid evaluation technique for fisheries integrating ecological and human dimensions." *Journal of Fish Biology* 83 (4):865-889. doi: https://doi.org/10.1111/jfb.12122.

Polbo das Rias. 2021. "Polbo das Rias Baixas Galegas." Polbo das Rias pesca artesanal, accessed 21/12/2021. https://polbodasrias.com/que-e/.

Principado de Asturias. 2021. Boletín Oficial del Principado de Asturias. https://sede.asturias.es/bopa/2021/12/03/2021-10354.pdf: Gobierno del Principado de Asturias.

Puig, Juan Juega. 2012. "El comercio maritimo en Galicia 1525- 1640." *Obradoiro de Historia Moderna* 21:1-5-130.

Pulponor S. Coop. Galega. 2022. "Inicio." accessed 30/11/2022. http://www.pulponor.com/web/index.php/gl-es/.

Roa-Ureta, Rubén H, M del Pino Fernández-Rueda, José Luis Acuña, Antonella Rivera, Ricardo González-Gil, and Lucía García-Flórez. 2021. "Estimation of the spawning stock and recruitment relationship of Octopus vulgaris in Asturias (Bay of Biscay) with generalized depletion models: implications for the applicability of MSY." *ICES Journal of Marine Science* 78 (6):2256-2270. doi: https://doi.org/10.1093/icesjms/fsab113.

Robin, Jean-Paul, Ana Moreno, Catalina Perales-Raya, Ignacio Sobrino, Angel Gonzalez, Carlos Montero, and Graham Pierce. 2021. Annexe 2 to WP4.2 Fisheries Summaries. Fisheries for Octopus vulgaris in the INTERREG Atlantic Area. https://www.cephsandchefs.com/wp-content/uploads/2021/06/WP4_2_FisheriesSummaries_Annex2_Octopus_fisheries_details.pdf: Cephs and Chefs.

Rocliffe, Steve, and Alberto Martin. 2020. "Charting a sustainable path. How the world's first MSC certified octopus fishery earned its spurs." 28/01/2022. http://astorias-octopus-stories.msc.org/.

Rosa de los Vientos. 2018. "Nueva Línea de Fabricación." accessed 01/12/2021. https://www.rosadelosvientos.es/nueva-linea-de-fabricacion/.

Rosa de los Vientos. 2021. "Inicio." Rosa de los Vientos, accessed 21/12/2021. https://www.rosadelosvientos.es/.

Surís-Regueiro, Juan C., and Jose L. Santiago. 2014. "Characterization of fisheries dependence in Galicia (Spain)." *Marine Policy* 47:99-109. doi: https://doi.org/10.1016/j.marpol.2014.02.006.

UNCTAD. 2021b. "Membership of UNCTAD and of the Trade and Development Board." United Nations Conference on Trade and Development, accessed 24/11/2021. https://unctad.org/about/membership.

United Nations. 2021b. "United Nations Treaty Collection - UNCLOS." accessed 24/11/2021. https://treaties.un.org/pages/ViewDetailsIII.aspx?src=TREATY&mtdsg_no=XXI-6&chapter=21&Temp=mtdsg3&clang=_en#EndDec.

Villasante, Sebastian, Gonzalo Macho, Susana Rivero Rodriguez, J Isusi de Rivero, Esther Divovich, Sarah Harper, Dirk Zeller, and Daniel Pauly. 2016. *Global atlas of marine fisheries: A critical appraisal of catches and ecosystem impacts.* Washington DC, USA: Island Press.

Villasante, Sebastian, Ana Tubío, Gillian Ainsworth, Pablo Pita, Manel Antelo, and José María Da-Rocha. 2021b. "Rapid Assessment of the COVID-19 Impacts on the Galician (NW Spain) Seafood Sector." *Frontiers in Marine Science* 8. doi: https://doi.org/10.3389/fmars.2021.737395.

WTO. 2021. "Spain and the WTO." World Trade Organization, accessed 24/11/2021. https://www.wto.org/english/thewto_e/countries_e/spain_e.htm.

Xunta de Galicia. 2014. Decreto 8/2014, de 16 de enero, por el que se regulan las cofradías de pescadores de Galicia y a sus federaciones. In *DOG no 19*, edited by Xunta de Galicia. https://www.xunta.gal/dog/Publicados/2014/20140129/AnuncioG0165-220114-0009_es.html

Xunta de Galicia. 2021a. "Galicia Calidade." accessed 01/12/21. https://www.galiciacalidade.gal/ES/home.

Xunta de Galicia. 2021b. "pescadeRías, ¿de onde se non?", accessed 01/12/21. https://deondesenon.xunta.gal/es.
